# Signatures of copy number alterations in human cancer

**DOI:** 10.1038/s41586-022-04738-6

**Published:** 2022-06-15

**Authors:** Christopher D. Steele, Ammal Abbasi, S. M. Ashiqul Islam, Amy L. Bowes, Azhar Khandekar, Kerstin Haase, Shadi Hames-Fathi, Dolapo Ajayi, Annelien Verfaillie, Pawan Dhami, Alex McLatchie, Matt Lechner, Nicholas Light, Adam Shlien, David Malkin, Andrew Feber, Paula Proszek, Tom Lesluyes, Fredrik Mertens, Adrienne M. Flanagan, Maxime Tarabichi, Peter Van Loo, Ludmil B. Alexandrov, Nischalan Pillay

**Affiliations:** 1 Research Department of Pathology, Cancer Institute, University College London, London, UK; 2 Department of Cellular and Molecular Medicine, UC San Diego, La Jolla, CA USA; 3 Department of Bioengineering, UC San Diego, La Jolla, CA USA; 4 Moores Cancer Center, UC San Diego, La Jolla, CA USA; 5 Cancer Genomics Laboratory, The Francis Crick Institute, London, UK; 6 CRUK–UCL Cancer Institute Translational Technology Platform (Genomics), London, UK; 7 Research Department of Oncology, UCL Cancer Institute, London, UK; 8 Genetics and Genome Biology, The Hospital for Sick Children, Toronto, Ontario Canada; 9 Institute of Medical Science, University of Toronto, Toronto, Ontario Canada; 10 Department of Laboratory Medicine and Pathobiology, University of Toronto, Toronto, Ontario Canada; 11 Department of Paediatric Laboratory Medicine, The Hospital for Sick Children, Toronto, Ontario Canada; 12 Division of Hematology/Oncology, The Hospital for Sick Children, Toronto, Ontario Canada; 13 Department of Paediatrics, University of Toronto, Toronto, Ontario Canada; 14 Translational Epigenetics, Division of Molecular Pathology, Institute of Cancer Research, London, UK; 15 Clinical Genomics, Translational Research Laboratory, Royal Marsden NHS Trust, London, UK; 16 Division of Clinical Genetics, Department of Laboratory Medicine, Lund University, Lund, Sweden; 17 Department of Clinical Genetics and Pathology, Division of Laboratory Medicine, Lund, Sweden; 18 Department of Cellular and Molecular Pathology, Royal National Orthopaedic Hospital NHS Trust, Stanmore, UK; 19 Institute for Interdisciplinary Research, Université Libre de Bruxelles, Brussels, Belgium

**Keywords:** Cancer genomics, Genome informatics

## Abstract

Gains and losses of DNA are prevalent in cancer and emerge as a consequence of inter-related processes of replication stress, mitotic errors, spindle multipolarity and breakage–fusion–bridge cycles, among others, which may lead to chromosomal instability and aneuploidy^1,2^. These copy number alterations contribute to cancer initiation, progression and therapeutic resistance^3–5^. Here we present a conceptual framework to examine the patterns of copy number alterations in human cancer that is widely applicable to diverse data types, including whole-genome sequencing, whole-exome sequencing, reduced representation bisulfite sequencing, single-cell DNA sequencing and SNP6 microarray data. Deploying this framework to 9,873 cancers representing 33 human cancer types from The Cancer Genome Atlas^6^ revealed a set of 21 copy number signatures that explain the copy number patterns of 97% of samples. Seventeen copy number signatures were attributed to biological phenomena of whole-genome doubling, aneuploidy, loss of heterozygosity, homologous recombination deficiency, chromothripsis and haploidization. The aetiologies of four copy number signatures remain unexplained. Some cancer types harbour amplicon signatures associated with extrachromosomal DNA, disease-specific survival and proto-oncogene gains such as *MDM2*. In contrast to base-scale mutational signatures, no copy number signature was associated with many known exogenous cancer risk factors. Our results synthesize the global landscape of copy number alterations in human cancer by revealing a diversity of mutational processes that give rise to these alterations.

## Abstract (web summary)

A new framework enables a pan-cancer reference set of copy number signatures derived from allele-specific profiles from different experimental assays.

## Main

Beyond alterations to single chromosomes, changes in genomic copy number can also occur through whole-genome doubling (WGD) and chromothripsis. WGD is when the entire chromosomal content of a cell is duplicated^7^ from a diploid to a tetraploid state, whereas chromothripsis is a ‘genomic catastrophe’ that leads to clustered rearrangements associated with oscillating copy number patterns^8^. These evolutionary events may occur multiple times at different intensities during tumour development and lead to highly complex cancer genomes^9^.

Previously, we developed a computational framework that enables the separation of somatic mutations into mutational signatures of single base substitutions (SBSs), doublet base substitutions (DBSs), and small insertions or deletions (IDs)^10,11^. Analyses of mutational signatures have provided unprecedented insights into the exogenous and endogenous processes that mould cancer genomes at a single nucleotide level^12^. Prior studies have also examined signatures of genomic rearrangements in cancer, and these have revealed insights into cancer-subtype-specific homologous recombination deficiency (HRD) and templated insertions^13,14^. Moreover, advancement in the bioinformatics integration of single nucleotide mutations, rearrangements and microsatellite instability profiles have improved signal-to-noise ratios to identify cancer processes^15^. However, rearrangement signatures can only be derived from whole-genome sequencing (WGS) data, which significantly limits their translational usability.

We recently developed a ‘mechanism-agnostic’ approach to summarize allele-specific copy number profiles in whole-genome sequenced sarcomas^16^, whereby a priori information on the mutational processes active in those cancers was not known, which we term copy number signatures. Other cancer-subtype-specific methods to interrogate copy number patterns that use known hallmarks of genomic instability have been applied to multiple myeloma^17^, breast cancer^18^, ovarian cancer^19^ and prostate cancer^20^. To our knowledge, there is currently no approach that allows the interrogation of copy number signatures derived from allele-specific profiles across multiple cancer types and across different experimental assays. To address this gap, we developed a new framework to decipher copy number signatures across cancer types (Supplementary Table 1) and multiple experimental platforms.

## A framework for copy number signatures

**Fig. 1:**
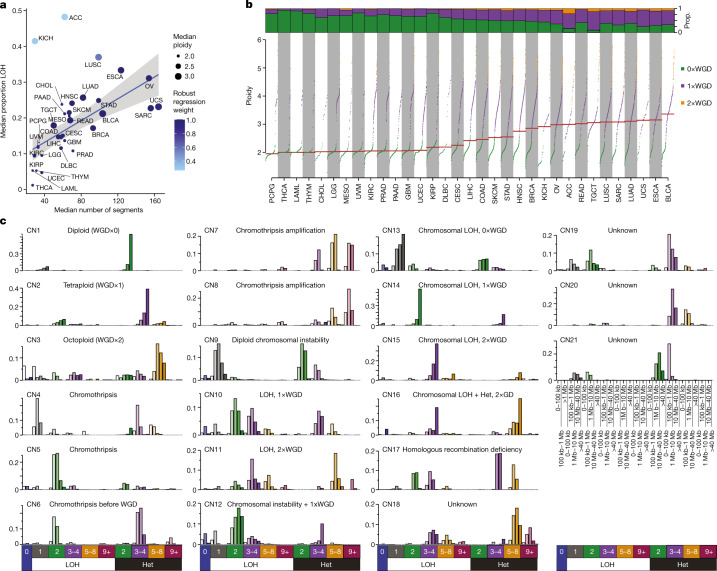
Pan-cancer copy number features of 33 tumour types from TCGA. **a**, Median number of segments in a copy number (CN) profile (*x* axis), median proportion of the genome that shows LOH (*y* axis) and the proportion of samples that have undergone one or more WGD events (size). The line of best fit from a robust linear regression is shown, whereby the colour of points indicates the weight of the tumour type in the regression model. Error bands indicate the 95% confidence interval﻿, *n* = 33, *t* = 4.95, *P* = 2.5e-5. See Supplementary Table 1 for cancer type abbreviations. **b**, Ploidy characteristics of all samples split by tumour type. Bottom, ploidy (*y* axis) for each sample in a tumour type (*x* axis), whereby samples are coloured by their genome doubling status as follows: 0×WGD, non-genome-doubled (green); 1×WGD, genome doubled (purple); and 2×WGD, twice genome-doubled (orange). Top, proportion (Prop.) of samples in each tumour type that are 0, 1 or 2×WGD. Horizontal lines indicate median ploidies. **c**, Decomposition plots of 21 pan-cancer copy number signatures (CN1–CN21). Heterozygosity (Het) status and total copy number (0–9+) are indicated below each column. Segment sizes are shown on the bottom right. Increasing saturation of colour indicates increasing segment size.

**Extended Data Fig. 1:**
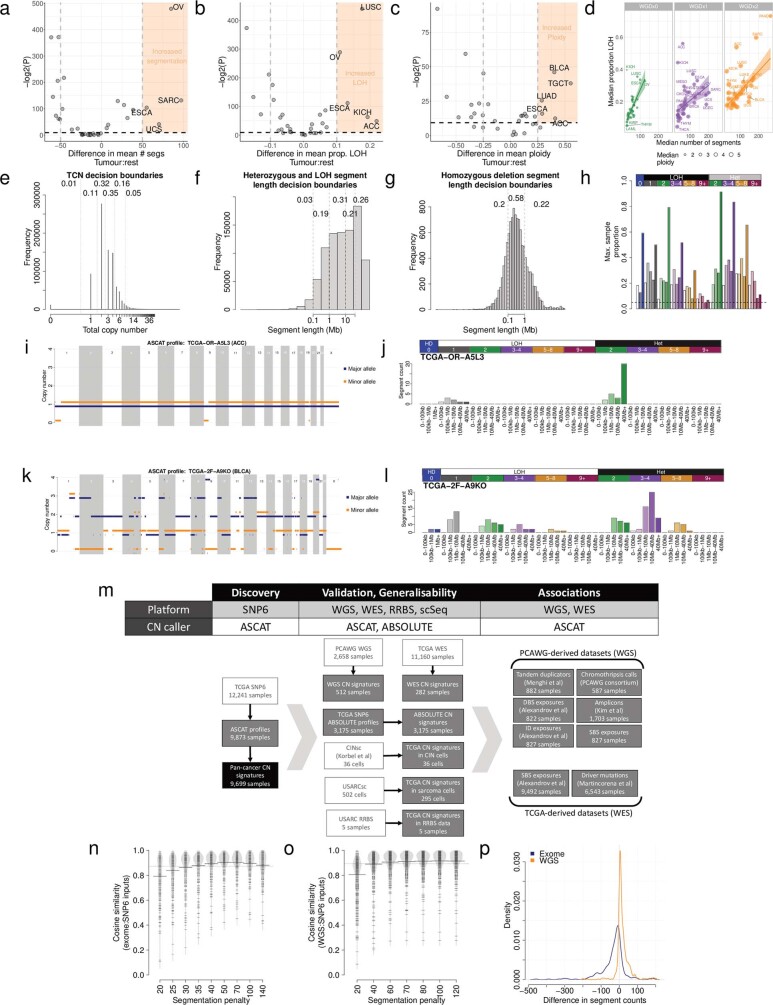
Choice of copy number categories. a) Enrichment of segment counts in TCGA tumour types: *x*-axis=difference in mean segment counts between tumour type and all other tumours, *y*-axis=-log2(*P*-value) from a two-sided Mann-Whitney test. b) Enrichment of LOH in TCGA tumour types: *x*-axis=difference in mean proportion of genome LOH between tumour type and all other tumours, *y*-axis=-log2(*P*-﻿value) from a two-sided Mann-Whitney test. c) Enrichment of high ploidy in TCGA tumour types: *x*-axis=difference in mean ploidy between tumour type and all other tumours, y-axis=-log2(*P*-value) from a two-sided Mann-Whitney test. d) Relationship between median number of segments (x-axis), median proportion of the genome that is LOH (*y*-axis) and ploidy (size) of 33 cancer types in TCGA, split by genome doubling status (panels). Error bands indicate the 95% confidence interval. e) Distribution of total copy number across TCGA. Dashed lines indicate decision boundaries between copy number classes. Numbers indicate the proportion of segments across TCGA that fall within the designated category. f) Maximum proportion of segments (*y*-axis) of each copy number category (*x*-axis) in any sample across TCGA. Increasing colour saturation indicates increasing segment length. g) Allele-specific copy number profile from a majority diploid sample (sample ID: TCGA-OR-A5L3, tumour type: ACC). Copy number (*y*-axis) across the genome (*x*-axis) is given for both the major (blue) and minor (orange) allele. i) Allele-specific copy number profile for a highly copy number aberrant sample (sample ID: TCGA-2F-A9KO, tumour type: BLCA). j) Copy number summary for TCGA-2F-A9KO. k) Overview of the discovery and validation datasets and samples used to develop the pan-cancer copy number signatures. Raw sequencing or array datasets that were used to generate copy number profiles are shown in white, previously processed datasets are shown in grey, and the pan-cancer copy number signature dataset is shown in black. WGS=whole genome sequencing, WES=whole exome sequencing, RRBS=reduced representation bisulfite sequencing, scSeq=single cell DNA sequencing. Throughout, samples have been excluded from analysis for data quality reasons, and to ensure sample matching between disparate datasets (see Methods for full details). l) Cosine similarity (y-axis) between input copy number summary vectors for exome sequencing and SNP6 array derived copy number profiles. m) Cosine similarity (y-axis) between input copy number summary vectors for whole genome sequencing and SNP6 array derived copy number profiles. n) Difference in segment counts between SNP6 array copy number profiles and whole genome sequencing (orange) or exome sequencing (blue) copy number profiles.

The extent of genomic instability—as measured through the number of copy number segments, the proportion of the genome displaying loss of heterozygosity (LOH) and the status of genome doubling—varied greatly among cancer types in The Cancer Genome Atlas (TCGA) (Fig. 1a, b). Nevertheless, a linear relationship was observed between the number of segments and the proportion of genomic LOH, which varies from cancers with diploid and copy number ‘quiet’ genomes (for example, acute myeloid leukaemia, thymoma and thyroid carcinoma; Fig. 1a and see Supplementary Table 1 for abbreviations of the cancer type) to cancers with highly aberrant copy number profiles (for example, high-grade serous ovarian carcinomas and sarcomas; Extended Data Fig. 1a, b). This linear relationship failed to hold only for adrenocortical carcinoma and chromophobe renal cell carcinoma, both of which demonstrated enrichment of LOH without enrichment of copy number segmentation (Extended Data Fig. 1a–c). In addition, considerable variability of ploidy was observed both between and within cancer types (Fig. 1b and Extended Data Fig. 1d).To distil this copy number heterogeneity and to capture biologically relevant copy number features, we developed a classification framework that encodes the copy number profile of a sample by summarizing the counts of segments into a 48-dimensional vector on the basis of the total copy number (TCN), the heterozygosity status and the segment size (Methods and Extended Data Fig. 1e–l).

To ensure the generalizability of our framework across platforms, we optimized the copy number calling strategy for each platform, which yielded a strong concordance of summary vectors between WGS, whole-exome sequencing (WES) and SNP6-profiling-derived copy number profiles (Extended Data Fig. 1m–p, Supplementary Table 1 and Methods).

## Repertoire of copy number signatures

Copy number matrices (*n* = 9,873; Supplementary Table 1) were decomposed using our previously established and extensively validated approach for deriving a reference set of signatures^10,11^ (Methods). This approach enabled the identification of both the shared patterns of copy number across all examined samples and the quantification of the number of segments attributed to each copy number signature in each sample, which we termed ‘signature attribution’.

**Extended Data Fig. 2:**
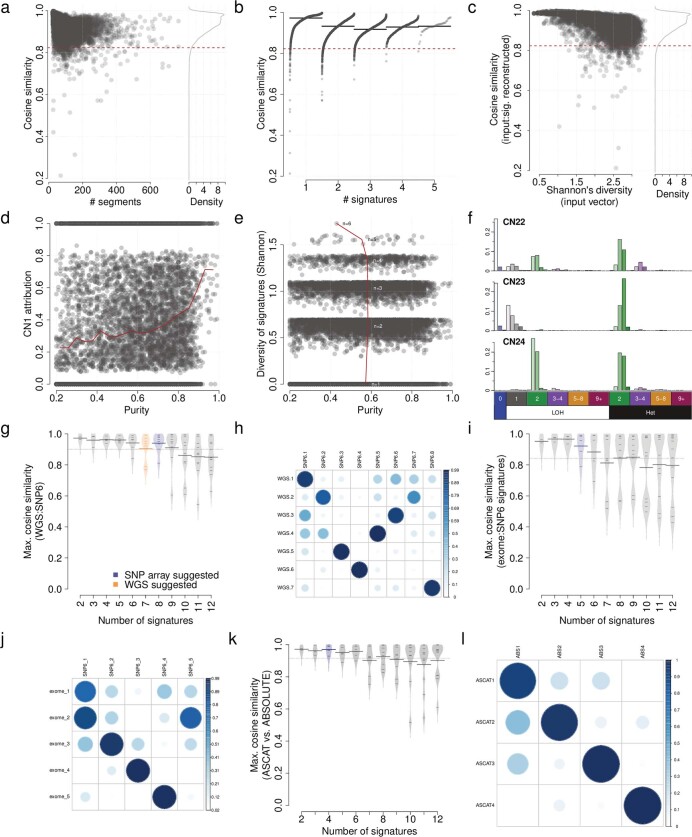
Signature derivations. a) Cosine similarity between input copy number 48 dimensional vectors, and signature reconstructed 48 dimensional vectors (*y*-axis) against number of segments in each copy number profile (*x*-axis). Dashed line indicates cosine similarity threshold for non-random similarity (*P* < 0.05). b) Cosine similarity between input copy number 48 dimensional vectors, and signature reconstructed 48 dimensional vectors (y-axis) against the number of signatures assigned in each sample (x-axis). Dashed line indicates cosine similarity threshold for non-random similarity (﻿*P* < 0.05). Solid lines indicate median cosine similarity. The number of signatures is plotted offset by the quantile of the sample. c) Cosine similarity between input copy number 48 dimensional vectors, and signature reconstructed 48 dimensional vectors (*y*-axis) against the Shannon’s diversity of copy number states in input 48 dimensional vector (*x*-axis). Dashed line indicates cosine similarity threshold for non-random similarity (*P* < 0.05). d) Relationship between tumour purity (*x*-axis) and CN1 attribution (*y*-axis). If purity was a confounding factor for copy number calling, purity would be positively associated with CN1 attribution due to a reduced power to call copy number alterations, however, the opposite relationship is seen here. e) Relationship between tumour purity (*x*-axis) and Shannon’s diversity of attributed copy number signatures (*y*-axis). If purity was a confounding factor for copy number calling, purity might be expected to negatively associate with diversity due to reduced power to call copy number alterations, however, no such association is seen here. f) Three artefactual signatures identified in the TCGA pan-cancer analysis. Artefactual signatures are typified by a large number of homozygous deletions (top two), or small segment sizes of equal copy number in LOH and heterozygous segments (bottom). g) Maximum cosine similarities between each WGS signature and any SNP6 identified signatures (i.e. closest matching signature cosine similarity, *y*-axis) from 512 samples, with varying numbers of signatures decomposed (*x*-axis). h) Cosine similarities between WGS (*x*-axis) and SNP6 (*y*-axis) identified signatures from 512 samples, with a segmentation penalty of 70. i) Maximum cosine similarities between each exome signature and any SNP6 identified signatures (i.e. closest matching signature cosine similarity, *y*-axis) from 282 samples, with varying numbers of signatures decomposed (x-axis). j) Cosine similarities between exome and SNP6 identified signatures from 282 samples, with a segmentation penalty of 70 and suggested number of signatures extracted. k) Maximum cosine similarities between each ABSOLUTE-derived signature and any ASCAT-derived signatures (i.e. closest matching signature cosine similarity, *y*-axis) from 3,175 samples, with varying numbers of signatures decomposed (*x*-axis). l) Cosine similarities between ABSOLUTE-derived and ASCAT-derived signatures from 3,175 samples, with four signatures extracted in each dataset.

In this first iteration (Methods), we identified 21 distinct pan-cancer signatures (Fig. 1c and Supplementary Table 2). These signatures accurately reconstructed the copy number profiles of 97% of the examined TCGA samples (*q* value < 0.05; Methods). The remaining 3% were poorly reconstructed owing to a combination of a low number of segments and/or a high diversity of copy number states in the copy number profile or few operative signatures identified, and are unrelated to purity estimates (Extended Data Fig. 2a–e). The 21 copy number signatures (CN1–CN21) were carefully inspected and categorized into six groups on the basis of their most prevalent features. CN1 and CN2 are primarily defined by >40 Mb heterozygous segments with TCNs of 2 and 3–4 respectively. CN3 is characterized by heterozygous segments with sizes >1 Mb and TCNs between 5 and 8. CN4–CN8 each have segment sizes between 100 kb and 10 Mb but with different TCN or LOH states. CN9–CN12 each have numerous LOH components with segment sizes <40 Mb. CN13–CN16 have whole-arm-scale or whole-chromosome-scale LOH events (>40 Mb). CN17 consists of LOH segments with TCNs between 2 and 4 as well as heterozygous segments with TCNs between 3 and 8, each with segment sizes 1–40 Mb. CN18–CN21 exhibit complex patterns of copy number alterations that are uncommon but are seen in distinct cancer types. In addition, three signatures (CN22–CN24) indicative of copy number profile oversegmentation were identified (Extended Data Fig. 2f).

We also systematically examined copy number signatures derived from WGS, WES and SNP6 profiles of the same samples. The results from this analysis demonstrated a strong concordance between signatures identified through different platforms (median cosine similarity of >0.8) (Extended Data Figs. 1m and 2g–j, and Supplementary Table 2) and different copy number callers (median cosine similarity of 0.98) (Extended Data Fig. 2k–l and Supplementary Table 2).

## Transitional nature of copy number signatures

**Extended Data Fig. 3:**
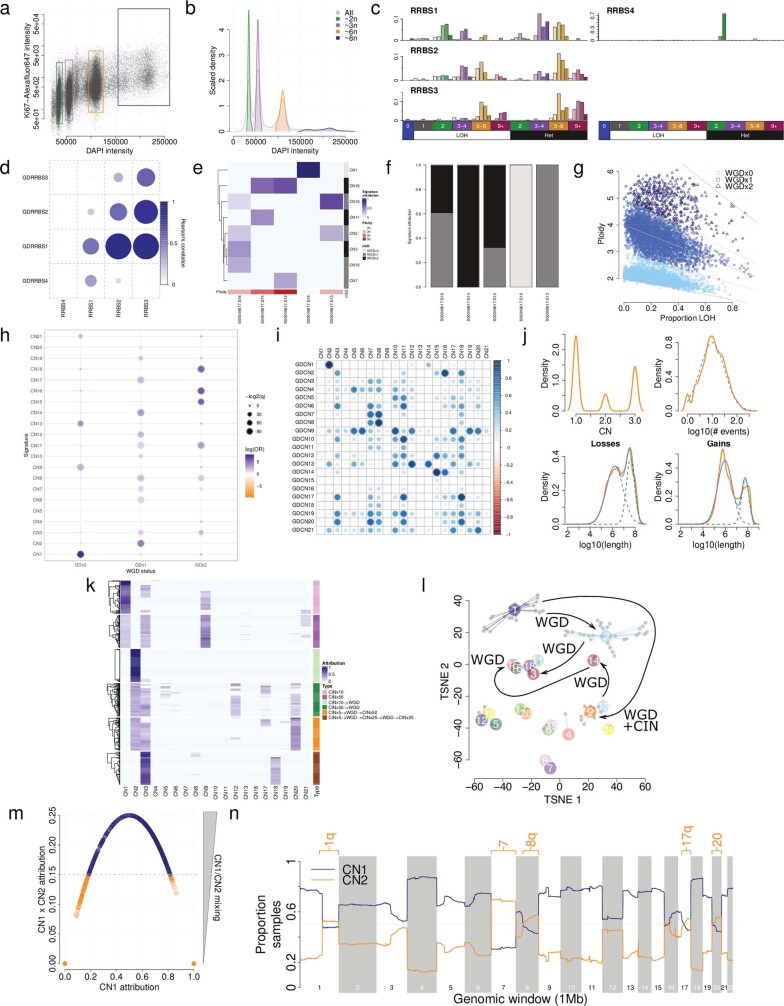
Ploidy associated signatures. a) Flow cytometry sorting of cells based on staining of DAPI (*x*-axis) as a proxy for DNA content and ki67 staining (*y*-axis) as a marker of proliferation. Cells were gated for sorting according to coloured boxes shown. b) Density of cells from flow sorting shown for all cells (grey) and for individual sorted populations of cells (coloured). c) De-novo signatures extracted from ploidy-sorted populations of cells profiled with reduced representation bisulfite sequencing. d) Cosine similarities between de-novo signatures and artificially genome- doubled versions of those signatures. Signature C has the highest similarity with genome doubled signature A, and signature B has the highest similarity with genome doubled signature C, indicating successive genome doublings leading to transitions of signatures. e) Attribution (blue) of pan-cancer signatures (*y*-axis) across ploidy-sorted populations of cells (*x*-axis). Ploidy of the sorted population is shown in red. Genome-doubling association of the pan-cancer signatures is shown in grayscale. f) Summed attribution of genome-doubling classifications of pan-cancer signatures across ploidy-sorted populations of cells. g) WGD calls for TCGA, based on ploidy and the proportion of the genome that is LOH. See Methods for details. WGDx0=non-genome doubled, WGDx1=genome doubled once, WGDx2=twice genome doubled. h) Associations between copy number signature exposure and WGD calls. GDx0=non-genome doubled, GDx1=genome doubled once, GDx2=twice genome doubled. i) Cosine similarities between signatures (CN1-21) and their artificially genome doubled counterparts (GDCN1-21). A high cosine similarity between e.g. CN2 and GDCN1 indicated that CN2 is a genome doubled version of CN1. j) Distributions of total copy number of segments (only TCN1-3, top-left), number of non-diploid segements (top-right), segment length of losses (TCN=1, bottom-left) and segment length of gains (TCN=3, bottom-right) for predominantly diploid (CN1+9>0.8) profiles in TCGA. Orange lines indicate empirical distributions, non-orange lines indicate simulated distributions. Dashed lines indicate components of mixture distributions, or the distribution for non-mixed distributions. Solid blue lines indicate joint distributions. k) Attributions (blue) of the 21 pan-cancer signatures (*x*-axis) in 6 simulation designs each of 100 samples (*y*-axis). CIN=random sub-chromosomal copy number gain or loss. WGD=whole genome doubling. l) TSNE representation of all non-artefactual signatures (coloured points). Inferences about the relationships between signatures (Extended Data Fig. 3) are indicated with arrows; WGD=whole-genome doubling, CIN=chromosomal instability. m) CN1 attribution (x-axis) against CN1 attribution × CN2 attribution in samples for which CN1+CN2 attribution = 1. Decision boundary for determining highly aneuploid samples is shown in grey. Orange points are taken for further analysis of aneuploidy. n) CN1 (blue) and CN2 (orange) recurrence (*y*-axis) across the genome (*x*-axis) in 472 highly aneuploid samples where CN1+CN2 attribution = 1. Chromosome arms with >50% samples attributed to CN2 are labelled.

The catalogue of somatic mutations in a cancer genome is the cumulative result of the mutational processes that have been operative over the lifetime of the cell of origin^21^. Analyses of SBS and ID mutational signatures have used assumptions and prior evidence that individual mutations are independent and additive^12^. However, this assumption is violated for large-scale macro-evolutionary events such as WGD^22^. Moreover, there are inherent challenges in inferring WGD using copy number calling algorithms that affect subclonal tumour reconstruction^23^. We therefore generated several synergistic lines of evidence to investigate the impact of WGD on copy number signatures. First, we undertook copy number profiling of experimentally ploidy-sorted populations of undifferentiated soft tissue sarcoma (Supplementary Table 3 and Extended Data Fig. 3a–f). Second, each copy number signature was tested for enrichment in non-, once- or twice-genome doubled samples (Extended Data Fig. 3g, h and Supplementary Table 3). Third, in silico simulations of genome doubling on the extracted signatures were performed (Methods, Extended Data Fig. 3i and Supplementary Table 3). Fourth, copy number profiles arising from dynamics of WGD and chromosomal instability (CIN) were simulated (Extended Data Fig. 3j) and re-examined for the previously derived signatures (Extended Data Fig. 3k and Supplementary Table 3).

By combining the preceding set of in silico simulations and wet-laboratory experiments, we confirmed the transitional nature of copy number signatures, with one signature being completely effaced by another after WGD (Extended Data Fig. 3l). In this model, a cancer with a diploid signature (CN1), may undergo WGD, which alters the signature CN1 into signature CN2. Alternatively, a cancer may show a CIN-transforming signature of CN1 into signature CN9. Through a combination of CIN and WGD, signature CN2 may transform into signature CN3. Meanwhile, CN13–CN15 are linked through successive WGD events on the background of early chromosomal losses.

Although WGD has a transitional effect on copy number signatures, we hypothesized that smaller scale events, such as segmental aneuploidy, may reflect additive behaviour similar to mutational signatures. To investigate this, we focused on the ploidy-associated signatures CN1 (diploid) and CN2 (tetraploid), for which an attribution of both signatures together indicates a hyperdiploid or subtetraploid profile (Extended Data Fig. 3m and Supplementary Table 3). We mapped these signatures across the cancer genomes so that only CN1 and CN2 were attributed (CN1 + CN2 = 1) and had a mixed attribution of those signatures (CN1 × CN2 > 0.15). This analysis recapitulated known patterns of aneuploidy in human cancer^24^, including gains of chromosomes 1q, 7, 8q, 16p, 17q and 20 in more than 50% of TCGA samples (Extended Data Fig. 3n).

## The landscape of copy number signatures

**Fig. 2:**
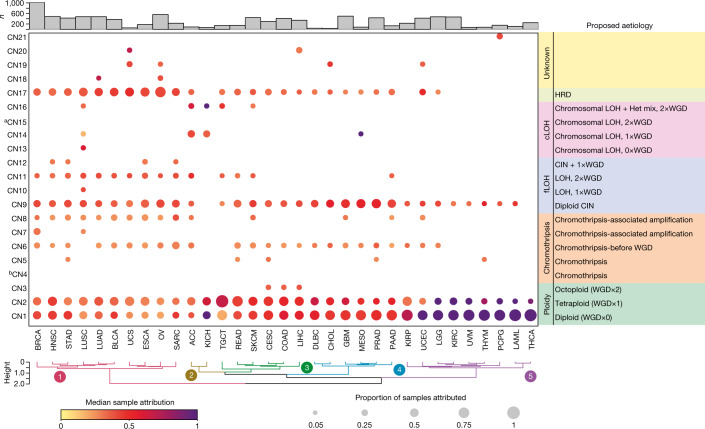
Distribution of copy number signatures across human cancers. Attributions of the 21 signatures (*y* axis) split by tumour type (*x* axis). The size of each dot represents the proportion of samples of each tumour type that shows the signature and the colour reflects the median attribution of the signature in each tumour type. Tumour/signature attributions with less than 5% of samples are not shown. Hierarchical clustering is shown below, sample sizes are shown above. ^a^CN15 was identified from an extraction of high LOH samples (>70% of the genome LOH), and is not found at ≥5% frequency in any tumour type. ^b^CN4 was identified in UVM at <5% frequency. Het mix, mixture of heterozygous segments.

Next, we surveyed the distribution of the 21 signatures across different cancer types (Fig. 2 and Supplementary Table 4). The ploidy-associated signatures CN1 and CN2 were found in most samples across all cancer types. Signatures CN4, CN7, CN10, CN18, CN20 and CN21 were derived through specific cancer type extractions and therefore unique to uveal melanoma, breast cancer, lung squamous carcinoma, ovarian carcinoma, liver cancer and paragangliomas, respectively. Signatures CN4–CN8 all showed segments of high TCNs and were seen in tumour types with known prevalent amplicon events^25^. CN9–CN12 showed differing patterns of hypodiploidy, with segment sizes of LOH < 40 Mb and WGD that was reflective of a type of structural CIN often induced by replication stress^26^. Signatures CN14 and CN16 were prevalent in adrenocortical carcinoma and chromophobe renal cell carcinoma, which indicates a link with the known patterns of chromosomal-scale LOH (cLOH) seen in these cancers^27,28^. Signature CN17 was prevalent in tumour types previously described as being HRD and enriched in the tandem duplicator phenotype (TDP)^29^. Different cancer lineages clustered together on the basis of the prevalence of signatures; namely TDP, WGD, diploid CIN, simple diploidy and cLOH (Fig. 2). This segregation of cancer types and their constituent signatures reflects the genomic heterogeneity imparted through WGD, chromothripsis and aneuploidy in human cancer^5,7^.

## Signatures associated with chromothripsis

**Fig. 3:**
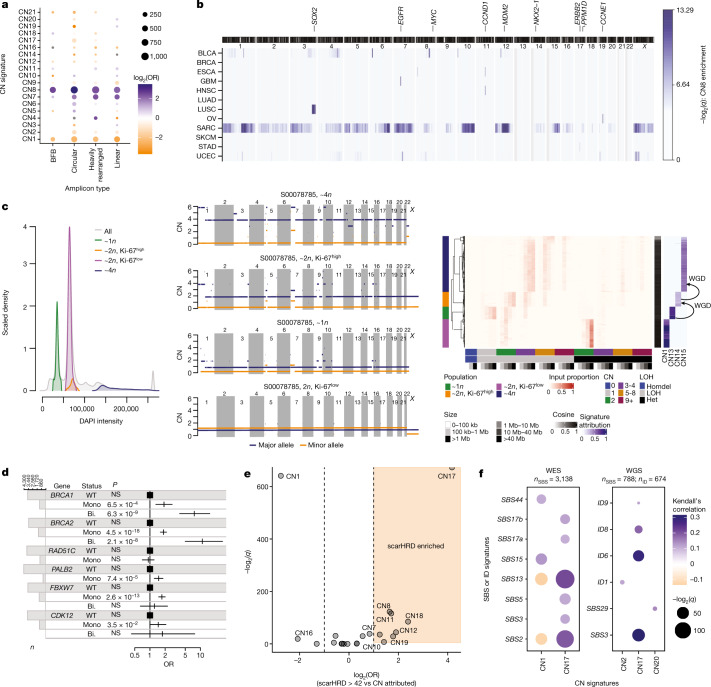
Biological inference of copy number signatures. **a**, Associations between signatures (*y* axis) and amplicon structures (*x* axis), displaying the *q* value (size) and log_2_(OR) (colour) from two-sided Fisher’s exact tests of genomic regions unattributed or attributed to each signature against each amplicon type. Only significant (*q* < 0.05) associations are shown. BFB, breakage–fusion–bridge. **b**, Enrichment of mapped CN8 in 1-Mb windows of the human genome across 8 cancer types in which ≥40 samples were attributed CN8. Colour indicates the –log_2_(*q* value) from a bootstrapping analysis to determine significance. An ideogram of chromosome bands is shown above. **c**, Single-cell sequencing from a near-genome-wide LOH undifferentiated soft tissue sarcoma. Sorted populations of cells based on ploidy and proliferation (left) were single-cell sequenced and copy number profiled (middle, representative cells). Copy number (*y* axis) across the genome (*x* axis) is given for both the major (blue) and minor (orange) allele. Copy number summaries (red) and signatures (blue) recapitulate the pattern seen in the copy number profiles (right). **d**, Association between mutational status of key HR pathway genes and CN17 attribution from a multivariate two-sided logistic regression model including cancer type as a covariate. NS, not significant (*P* ≥ 0.05). Squares represent point estimates for the odds ratio (OR). Horizontal lines indicate 95% confidence intervals. *n* = 4,919 biologically independent tumours. Bi., bi-allelic alteration; Mono., monoallelic alteration; WT, wild type. **e**, Association between signature attribution and scarHRD score, an orthogonal test for HRD, displaying –log_2_(*q*) (*y* axis) and log_2_(OR) (*x* axis) from two-sided Fisher’s exact tests in which scarHRD positivity was based on a threshold of >42. A half dot indicates an infinite –log_2_(*q* value) (*q* = 0). **f**, Correlation between copy number signature (*x* axis) attribution and SBS  or ID signature (*y* axis) exposure across TCGA exomes (left) and whole genomes (right). The strength of correlation is indicated by colour (orange, anticorrelated, blue, correlated), the *q* value is indicated by point size. Non-significant (*q* > 0.01) associations are not shown.

**Extended Data Fig. 4:**
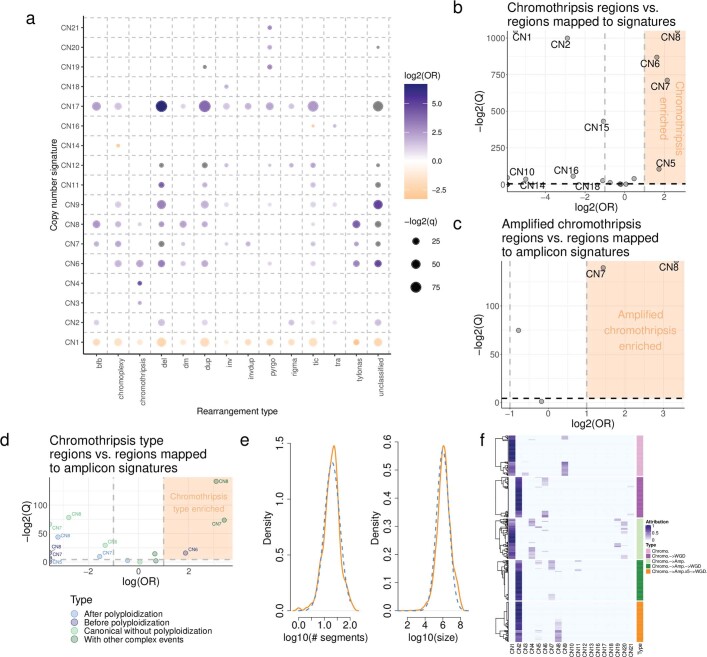
Chromothripsis-associated signatures. a) Associations between copy number signature attribution (y-axis) and rearrangement phenomena (x-axis) described in Hadi *et al*. (2020). Effect size (log2 odds ratio, colour), and significance level (-log2 *Q*-value, size) from a Fisher’s exact test are displayed. b) Correlation between copy number signature attributed segments and chromothriptic regions at a genomic level. *X*-axis=effect size (log odds ratio), *y*-axis=significance (-log2 *Q*-value). A half dot indicates an infinite value (﻿*Q* = 0, or OR=Inf). c) Same as for (a), but correlated against amplified chromothripsis. CN7-8 OR= OR = 2.69 and 10.08 respectively, *Q*﻿ < 0.05. d) Same as for (a), but correlated against distinct chromothripsis types. e) Distributions of the number of segments (left) and segment sizes (right) on chromothriptic chromosomes identified by PCAWG. Orange lines indicate empirical distributions. Blue dashed lines indicate simulation distributions. f) Attributions (blue) of the 21 pan-cancer signatures (*x*-axis) in 5 simulation designs each of 100 samples (*y*-axis). Chromo.=chromothripsis. WGD=whole genome doubling. Amp=single gain of the derivative chromothriptic chromosome.

Oncogene amplification is associated with aggressive behaviour in cancer^25^. Reasoning that signatures with high levels of TCN (CN4–CN8) could be associated with genomic amplification, we correlated these signatures with known classes of amplicons^25,30^. All amplicon signatures were positively associated with one or more amplicon types (Fig. 3a and Extended Data Fig. 4). CN8, which shows very high copy number states and is enriched in nine cancer types (two-sided Mann–Whitney test, *q* < 0.05), was strongly associated with all four classes of amplicons, although most strongly with extra-chromosomal circular DNA amplicons (ecDNA) and the recently described large amplicon phenotype termed ‘tyfonas’^31^ (Extended Data Fig. 4a).

Recent evidence shows that genomic amplification can evolve through inter-related processes of chromothripsis, breakage–fusion–bridge and ecDNA formation^32^. To test this finding, we mapped the copy number signatures with known regions of chromothripsis^33^ across the cancer genome (Methods), which revealed that CN5–CN8 are enriched in chromothriptic regions (Extended Data Fig. 4b and Supplementary Table 5). Each of these signatures was dominated by small segments, while CN7 and CN8 were both strongly associated with amplified chromothripsis^33^ (Extended Data Fig. 4c), larger DNA segments and complex chromothriptic events (Extended Data Fig. 4d). Simulations of copy number profiles incorporating processes of chromothripsis, WGD and chromosomal duplication (Extended Data Fig. 4e) demonstrated that CN4–CN8 can be generated through chromothripsis-like events. Moreover, these signatures reflected distinct life histories of tumours, such as chromothripsis before or after WGD (Extended Data Fig. 4d, f and Supplementary Table 5).

**Extended Data Fig. 5:**
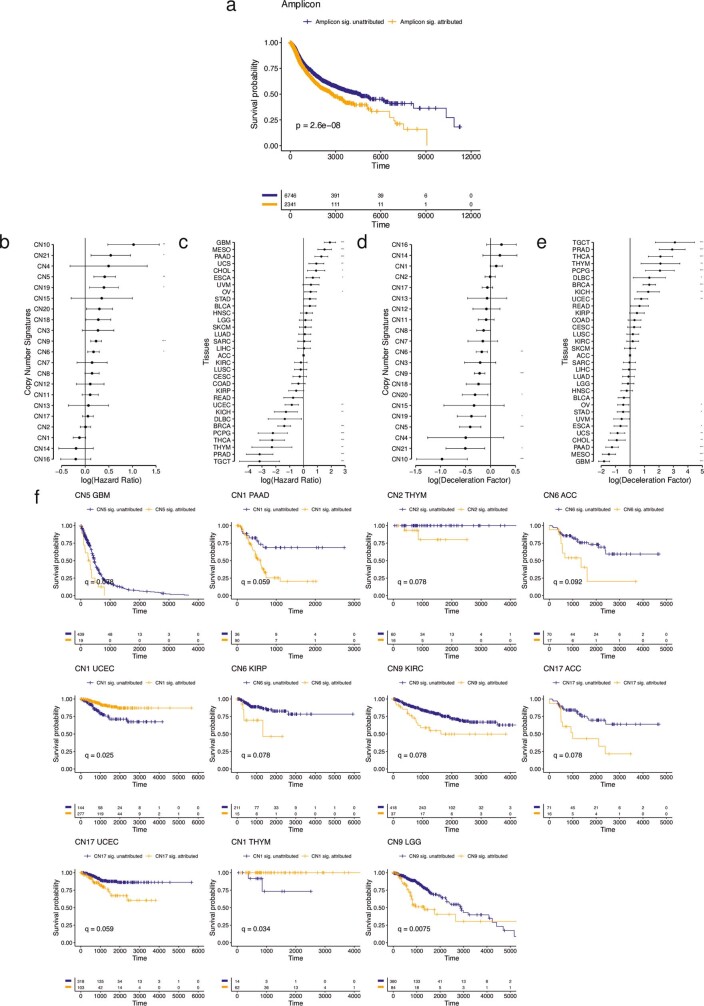
Survival associations. a) Kaplan-Meier curves of disease specific survival for patients whose tumours are amplicon signature (CN4:8) attributed (orange) and non-attributed (blue). b) Cox-model hazard ratios (*x*-axis) for copy number signatures (*y*-axis) with copy number signature attribution and tumour type as a covariates (see Extended Data Fig. 5e). Horizontal bars indicate 95% confidence intervals. Sample sizes are given in Supplementary Table 5. c) Cox-model hazard ratios (*x*-axis) for tumour types (*y*-axis) with copy number signature attribution (see Extended Data Fig. 5d) and tumour type as covariates. Horizontal bars indicate 95% confidence intervals. ACC is taken as the reference tumour type (square point). d) Accelerated failure time deceleration factors (*x*-axis) for copy number signatures (*y*-axis) with copy number signature attribution and tumour type as a covariates (see Extended Data Fig. 5c). A log(deceleration factor)<1 indicates reduced survival time (accelerated failure time), while a log(deceleration factor)>1 indicates increased survival time (deaccelerated failure time). Horizontal bars indicate 95% confidence intervals. Sample sizes are given in Supplementary Table 5. e) Accelerated failure time deceleration factors (x-axis) for tumour types (y-axis) with copy number signature attribution (see Extended Data Fig. 5b) and tumour type as covariates. A log(deceleration factor)<1 indicates reduced survival time (accelerated failure time), while a log(deceleration factor)>1 indicates increased survival time (deaccelerated failure time). Horizontal bars indicate 95% confidence intervals. ACC is taken as the reference tumour type (square point). f) Kaplan-Meier curves for within-tumour type associations with copy number signature attribution. Tumour type/copy number signature combinations with a significant effect on survival (﻿*Q* < 0.05) are displayed.

Chromothripsis and gene amplification are both independently associated with poor prognosis^25,34^. Attribution of any of the five amplicon signatures in their respective cancer types showed poor disease-specific survival in a univariate pan-cancer analysis (Extended Data Fig. 5a and Supplementary Table 5). Similarly, multiple amplicon signatures were associated with reduced disease-specific survival in multivariate pan-cancer and cancer subtype analyses, with consistent results from analyses based on Cox-model hazard ratios (Extended Data Fig. 5b, c and Supplementary Table 5) and analyses based on accelerated failure times (Extended Data Fig. 5d, e and Supplementary Table 5). For example, a cancer-type-specific survival analysis revealed that patients with glioblastoma with operative signature CN5 had poor disease-specific survival (172 days reduced median survival; Extended Data Fig. 5f and Supplementary Table 5). To determine the topographical localization of the amplification events, we mapped the most common amplicon signature with the highest amplification level, CN8, across the genome in eight cancer types (*n* ≥ 40 in each type) that were attributed CN8, and assessed CN8 enrichment in each cancer type through a bootstrapping analysis. This revealed cancer-type-specific enrichment of CN8 in regions harbouring oncogenes that are commonly amplified in their respective cancer types (Fig. 3b and Supplementary Table 5).

## Signatures associated with LOH

**Extended Data Fig. 6:**
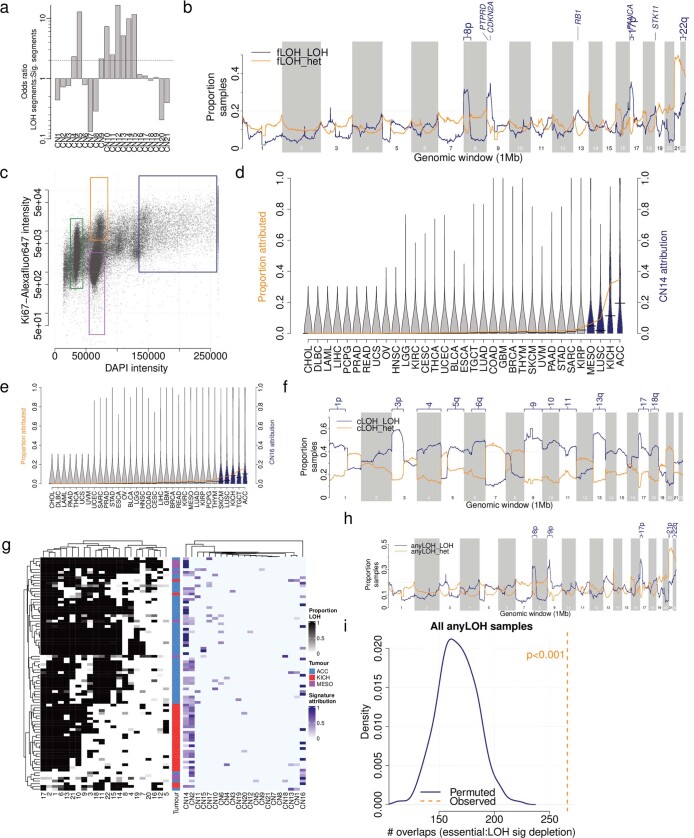
LOH associated signatures. a) Association between LOH segments and mapped copy number signature segments across the full TCGA cohort. b) Recurrence of mapped LOH signatures (*y*-axis) across the genome in 1Mb bins (*x*-axis), split by LOH (blue) or heterozygous (orange) segments. Tumour suppressor genes with >20% of samples with LOH signatures are labelled. c) FACS sorting of undifferentiated sarcoma cells. Cells were gated on DAPI staining intensity (*x*-axis, proxy for DNA content), and ki67 intensity (*y*-axis, indicating replicating cells). Gates were chosen to isolate population of near haploid cells (~1*n*, green), replicating and non- replicating ~2*n* populations of cells (orange and purple respectively) and a ~4*n* population of cells (blue). d) Prevalence (orange line) and distribution (violins) of CN14 attributions across TCGA cancer types. Blue violins are cancer types significantly enriched in CN14 compared to all others (﻿*Q* < 0.05, Mann Whitney test). KICH enrichment: OR = 4.6, ﻿*P* = 3.0e-3, Fisher’s exact test. ACC enrichment: OR = 8.9, ﻿*P* = 6.3e-9, Fisher’s exact test. e) Prevalence (orange line) and distribution (violins) of CN16 attributions across TCGA cancer types. Blue violins are cancer types significantly enriched in CN21 compared to all others (﻿*Q* < 0.05, Mann Whitney test). KICH enrichment: OR = 30.5, ﻿*P* = 1.0e-21, Fisher’s exact test. ACC enrichment: OR = 37.4, ﻿*P* = 3.5e- 33, Fisher’s exact test. f) Recurrence of mapped arm-level LOH signatures (*y*-axis) across the genome in 1Mb bins (*x*-axis), split by LOH (blue) or heterozygous (orange) segments. Chromsome arms with >50% of samples with LOH signatures are labelled. g) Left: Heatmap of LOH prevalence by chromosome (*x*-axis) and sample (*y*-axis) for all CN13-CN16 attributed ACC, KICH or MESO samples. Samples are clustered according to chromosomal LOH levels. Right: Copy number signature attributions for the same samples. h) Recurrence of mapped chromosomal-scale and focal LOH signatures (*y*-axis) across the genome in 1Mb bins (*x*-axis), split by LOH (blue) or heterozygous (orange) segments. Chromosome arms with >20% of samples with LOH signatures are labelled. i) Enrichment of essential genes in regions of the genome with >20% of the samples having heterozygous segments of cLOH or fLOH signatures through bootstrapping of genomic regions.

LOH is an important mechanism that contributes to the inactivation of tumour suppressor genes during cancer development^6,33,35^. Nine signatures were positively correlated with LOH regions of the genome (Extended Data Fig. 6a) and were recurrently found around known tumour suppressor genes (Extended Data Fig. 6b and Supplementary Table 6). Four of these signatures (CN9–CN12) exhibited predominantly small segment sizes and very few that were >40 Mb (Fig. 1c) and were therefore termed focal LOH (fLOH) signatures.

Genome-wide chromosomal-scale losses (near haploidy), often followed by genome doubling, are associated with poor prognosis in B-cell acute lymphoblastic leukaemia^36^. Conversely, haploidization is associated with immune cell infiltration and a relatively better prognosis in undifferentiated soft tissue sarcoma^16^. This is an uncommon event in cancer (0.2% prevalence in TCGA; Extended Data Fig. 3g) but is seen in as much as 3% of sarcomas and mesotheliomas. We reasoned that this phenomenon could result in a distinctive copy number signature that could have clinical implications. We selectively extracted signatures from cancers that display an LOH of more than 70% of the genome, which revealed the distinctive signatures CN13, CN14 and CN15. We experimentally confirmed these rare signatures through ploidy sorting and single-cell DNA sequencing (SCS) of undifferentiated soft tissue sarcoma (Extended Data Fig. 6c and Supplementary Table 6), which are known to have genome-wide LOH and a complex subclonal structure^16^. These unique signatures were represented in multiple subclones and reflected successive WGDs on a background of genome-wide LOH (Fig. 3c). Other patterns of distinctive hypodiploidy^37,38^ were enriched in adrenocortical carcinoma and chromophobe renal cell carcinoma (CN14 and CN16; Extended Data Fig. 6d, e). Mapping of these signatures to the genome displayed recurrent LOH in chromosome regions 1p, 3p, 5q, 9, 10q, 13q and 17p (Extended Data Fig. 6f, g and Supplementary Table 6), which matched known patterns of aneuploidy in these cancers^27,28^.

An allele-specific deletion of a DNA segment harbouring an essential gene that results in LOH represents a potential therapeutic vulnerability^39^, and such regions have been shown to be under strong negative selection for deleterious mutations^22,40,41^. We hypothesized that in cancers with extensive LOH signatures, regions of the genome with a high density of essential genes may show retention of heterozygosity. An enrichment analysis revealed that regions of retained heterozygosity were enriched in essential genes compared with random selections of regions across the genome (Extended Data Fig. 6h, i). These essential-gene-enriched regions are probably subject to strong negative selection for genomic losses and therefore represent a particularly rich area to explore for therapeutics. This is particularly relevant to cancers that have extensive LOH, as tagged here with cLOH signatures in adrenocortical carcinomas, kidney chromophobe cancers and mesotheliomas.

## Signatures associated with HRD

**Extended Data Fig. 7:**
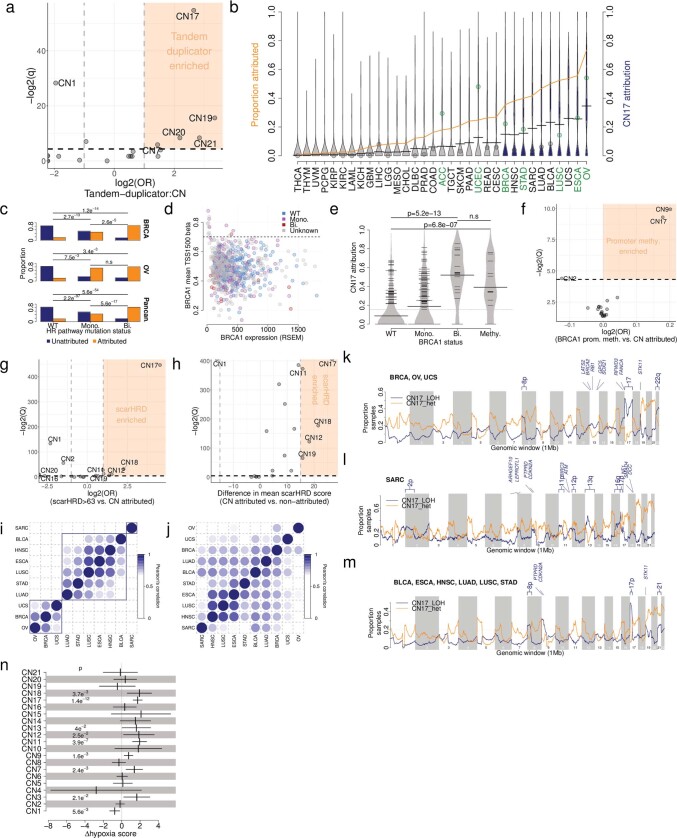
Signature of homologous recombination deficiency. a) Associations between copy number signature attributed samples and tandem-duplicator phenotype samples, displaying -log2(﻿*Q*-values) (y-axis) and log2 odds ratios (*x*-axis). CN17 association: OR = 6.3, ﻿*Q* = 3.6e-17, Fisher’s exact test. b) Prevalence (orange line) and distribution (violins) of CN17 attributions across TCGA cancer types. Blue violins are cancer types significantly enriched in CN17 compared to all others (﻿*Q* < 0.05, Mann Whitney test). Points indicate the prevalence of TDP in given tumour types from the literature (Menghi et al., 2018) coloured by over- (green) or underrepresentation (gray). A half dot indicates an infinite value. c) Correlation of CN17 attribution (*y*-axis) with mutational status of one or more genes of the homologous recombination pathway (*x*-axis) in breast cancer (top, *n* = 589), ovarian cancer (middle, *n* = 309) or pan-cancer (bottom, *n* = 4,919). WT=wild type. Mono = Mono-allelic and Bi = bi-allelic. Two-sided Fisher’s exact test: ﻿*Q*-values are given above, n.s.=﻿*Q* ≥ 0.05. d) Relationship between *BRCA1* gene expression (*x*-axis) and promoter methylation (*y*-axis). A mean TSS1500 beta cutoff of 0.7 was chosen to indicate promoter hyper-methylation, correlating with gene silencing. e) CN17 attribution (*y*-axis) split by *BRCA1* mutational status (*x*-axis) in TCGA breast cancers. WT=wild type (*n* = 220), Mono.=mono-allelic mutation (*n* = 148), Bi.=bi-allelic mutation (*n* = 19), Methy.=promoter hypermethylation (*n* = 13). Two-sided Mann-Whitney test: ﻿*P*-values are given above, n.s.=﻿*P* ≥ 0.05. f) Association between copy number signature attribution and promoter hypermethylation of *BRCA1* (beta > 0.7), displaying -log2(﻿*Q*-values) (*y*-axis) and log2 odds ratios (*x*-axis) from a multivariate logistic regression model with cancer type as a covariate. g) Association between copy number signature attribution and scarHRD score, displaying -log2(﻿*Q*-values) (*y*-axis) and log2 odds ratios (*x*-axis) from a Fisher’s exact test where scarHRD positivity was thresholded at >63. A half dot indicates an infinite value. h) Association between copy number signature attribution and scarHRD score, displaying -log2(﻿*Q*-values) (*y*-axis) and difference in mean scarHRD scores (*x*-axis) from a Mann-Whitney test on continuous scarHRD scores. A half dot indicates an infinite value. i) Pearson’s correlation of recurrence of mapping of LOH segments of CN17 to the genome calculated for all pairwise comparisons of CN17-enriched tumour types. j) Pearson’s correlation of recurrence of mapping of CN17 to the genome from pairwise comparisons of CN17 enriched tumour types for heterozygous segments. k) Recurrence of mapped CN17 in 1 Mb windows of the human genome in all CN17 attributed BRCA, OV and UCS samples, split by LOH (blue) and heterozygous segments (orange). Tumour-suppressor genes in regions with >20% samples attributed to CN17 with LOH segments are labelled. l) Recurrence of mapped CN17 in 1 Mb windows of the human genome in all CN17 attributed SARC samples, split by LOH (blue) and heterozygous segments (orange). Tumour-suppressor genes in regions with >20% samples attributed to CN17 with LOH segments are labelled. m) Recurrence of mapped CN17 in 1 Mb windows of the human genome in all CN17 attributed STAD, LUAD, BLCA, HNSC, ESCA and LUSC samples, split by LOH (blue) and heterozygous segments (orange). Tumour-suppressor genes in regions with >20% samples attributed to CN17 with LOH segments are labelled. n) Association between copy number signature (y-axis) attribution and hypoxia score (*x*-axis=effect size) in a two-sided multivariate logistic regression model including cancer type as a covariate. Vertical bars indicate effect estimates, horizontal bars indicate 95% confidence intervals. *P*﻿-values for significant associations (﻿*P* < 0.05) are given (non-significant values can be found in Supplementary Table 7). *n* = 6,805 biologically independent tumours.

Somatic tandem duplications (TDs) are commonly found in breast cancer and ovarian cancer that show failure of homologous recombination (HR) repair of DNA double-strand breaks, for example, owing to defective *BRCA1* or *BRCA2* expression^29,42^. A detailed characterization of TD across cancer types has revealed three patterns with duplicated segments that range around 10 kb, 200 kb or 2 Mb (ref. ^29^). CN17 has a segment size distribution that overlaps with the largest of these three patterns and was strongly associated with TD (Extended Data Fig. 7a and Supplementary Table 7; odds ratio (OR) = 6.3, *q* = 3.6 × 10^–17^, two-sided Fisher’s exact test) and enriched in cancer types known to show TD^29^ (Extended Data Fig. 7b).

We found an enrichment of CN17 in samples that harbour germline and/or somatic mutations in the key HR genes *BRCA1*, *BRCA2*, *PALB2*, *FBXW7* and *CDK12*, but not *RAD51C* (Fig. 3d and Supplementary Table 7), and in a more comprehensive analysis of the HR repair pathway (Extended Data Fig. 7c and Supplementary Table 7). In addition to mutations, epigenetic silencing of HR genes can result in HRD^43^. This was further investigated by examining the promoter methylation status of *BRCA1* in breast cancers with CN17 attribution. This revealed levels of CN17 comparable to samples with bi-allelic loss of HRD genes (Extended Data Fig. 7d, e). Extending this to a multivariate pan-cancer analysis showed that CN17 was significantly associated with promoter hypermethylation of *BRCA1* across cancer types (Extended Data Fig. 7f), in addition to CN9. Further supporting the link between CN17 and HRD, other lines of evidence, including scarHRD scores^44^ and SBS and ID mutational signatures from WES and WGS, showed a strong correlation with CN17 attribution (Fig. 3e, f, Extended Data Fig. 7g, h and Supplementary Table 7). In addition, positive associations were found between CN17 and the APOBEC mutational signatures SBS2 and SBS13, which are prevalent around DNA double-strand breaks^45^.

Genome topographical mapping of CN17 in CN17-enriched cancers revealed a distribution of LOH segments (Extended Data Fig. 7i) that was tumour-type-specific, a feature not seen in heterozygous segments (Extended Data Fig. 7j), which suggests that there is tissue-specific-selective forces associated with DNA deletions. Breast cancer, ovarian cancer and uterine carcinosarcoma displayed recurrent chromosomal LOH at 8p, 17 (including *BRCA1* and *TP53*) and 22 (Extended Data Fig. 7k). Focal LOH was also observed on 9q around *TSC1*, 13q around *BRCA2* and *RB1*, and 19p around *STK11*. By contrast, CN17-attributed sarcomas displayed strong peaks of recurrent LOH around known sarcoma tumour suppressor genes^46^ (*CDKN2A*, *RB1* and *TP53*; Extended Data Fig. 7l). The six other tumour types enriched in CN17 displayed recurrent chromosomal LOH at 8p, 9p, 17p, 19p and 21 (Extended Data Fig. 7m). These findings suggest that copy number signatures could be helpful in revealing the potential mechanisms that underpin the positive selection of cancer genes.

We hypothesized that tumour microenvironmental conditions could provide an explanation for finding CN17 in cancers without mutations in HRD-related genes, as hypoxia can fuel HRD in many cancers^47,48^. Modelling of copy number signature attributions with comprehensive readouts of transcriptome-based hypoxia gene signatures across cancer types^49^ revealed a significant positive correlation with CN17 attribution and with signatures of aneuploidy. This result confirms that hypoxia is strongly associated with different patterns of genomic instability, including HRD, in cancer genomes (Extended Data Fig. 7n).

## Signatures associated with cancer-driver genes

**Fig. 4:**
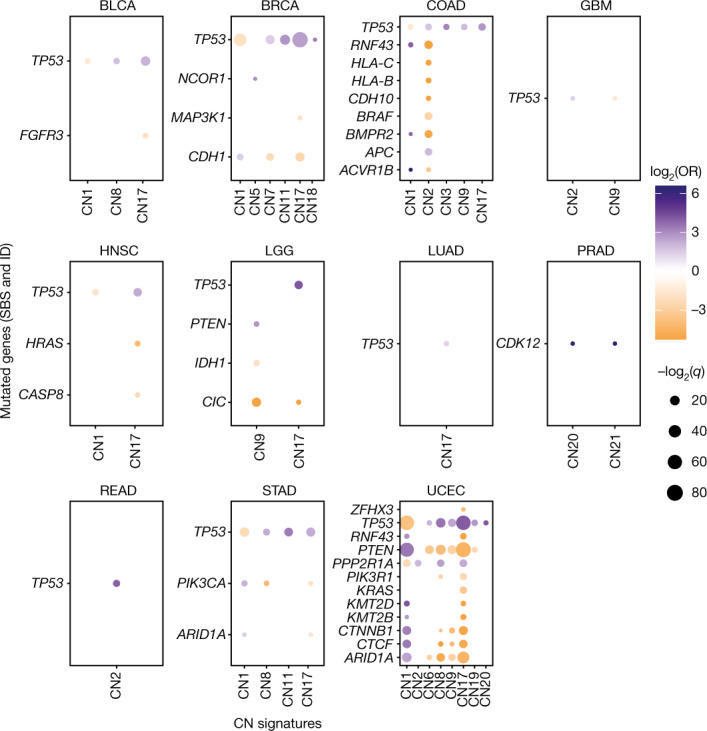
Genomic associations of copy number signatures. Associations between copy number signatures (*x* axis) and driver-gene single nucleotide variant and ID status (*y* axis) across each TCGA tumour type (panels). Effect size (log_2_(OR), colour), and significance level (–log_2_(*q*), size) from two-sided Fisher’s exact tests are displayed. Non-significant (*q* ≥ 0.05) associations are not shown.

**Extended Data Fig. 8:**
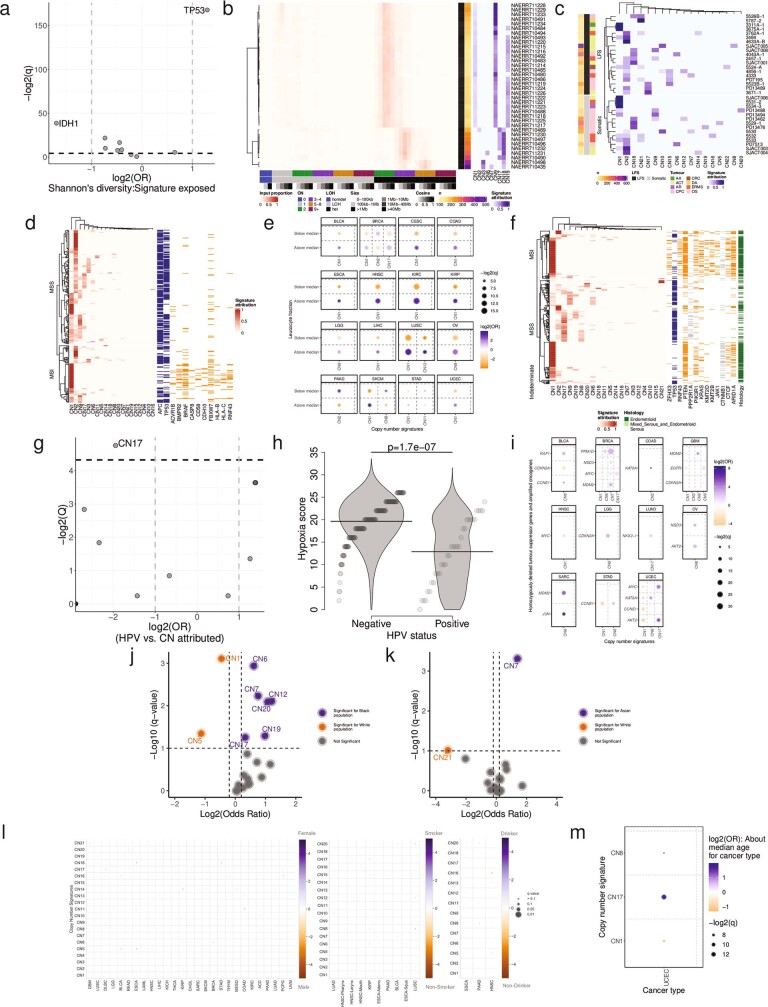
Genomic and clinical correlates. a) Correlation between Shannon’s diversity index of signature proportions in samples, and driver gene mutation status. Effect size (log2 odds ratio, *y*-axis) and significance (-log2 ﻿*Q*-value, *y*-axis) are displayed. Driver genes with |log2(OR)|>1 and ﻿*Q* < 0.05 are labelled. TP53 association: OR = 3.65, ﻿*Q* = 3.0e- 51. b) Pan-cancer copy number signature attribution in 36 *TP53* mutant RPE1 single cell sequenced cells (Mardin *et al*., 2020). Left: input profile summaries (red). Right: copy number signature attribution (blue). c) Heatmaps of copy number signatures identified across the spectrum of Li-Fraumeni Syndrome (LFS) associated cancers and somatic *TP53* mutant cancers. Colour indicates the strength of signature attribution. Somatic=somatic *TP53* mutant cancers, LFS=germline *TP53* mutant cancers. d) Heatmap of copy number signature attribution (left) and driver gene mutation status (right) for all COAD samples, split by microsatellite instability status. Driver gene mutations are coloured orange or blue for genes that are positively (OR > 1, ﻿*Q* < 0.05) or negatively (OR < 1, ﻿*Q* < 0.05) associated with MSI status respectively, and grey for genes that are not associated with MSI status (q≥0.05). Association between CN1 or CN2 and MSI status: OR = 1.8 and 0.21, ﻿*P* = 0.03 and 7.7e-9 respectively, Fisher’s exact test. e) Correlations between leukocyte fraction (*y*-axis, split by median value per tumour type) and copy number signature attribution (*x*-axis). Effect size given as log2(OR) (colour) and significance given as ﻿*Q*-values (size) are displayed. Only associations with |log2(OR)|>1 and ﻿*Q* < 0.05 are shown. Associations were tested with a logistic regression model with leukocyte fraction as the dependent variable and tumour purity and copy number signature attribution (binarized) as independent variables (purity associations not shown). f) Heatmap of copy number signature attribution (left) and driver gene mutation status (right) for all UCEC samples, split by microsatellite instability status. Driver gene mutations are coloured orange or blue for genes that are positively (OR > 1, ﻿*Q* < 0.05) or negatively (OR < 1, ﻿*Q* < 0.05) associated with MSI status respectively, and grey for genes that are not associated with MSI status (q≥0.05). Association between CN1 or CN2 and MSI status: OR = 0.17 and 2.6, ﻿*P* = 1.1e-10 and 7.0e-4 respectively, Fisher’s exact test. g) Association between HPV status and copy number signature attribution. *X*-axis=effect size (log odds ratio), *y*-axis=significance (-log2 ﻿*Q*-value). Fisher’s exact test. A half dot indicates an infinite value. h) Association between hypoxia score (*y*-axis) and HPV status (*x*-axis). Two-sided Mann-Whitney test. *n* = 259 biologically independent tumour samples. i) Associations between copy number signatures (x-axis) and driver gene copy number alteration status (*y*-axis, amplification for oncogenes, homozygous deletion for tumour-suppressor genes) across each TCGA tumour type (panels). Effect size (log2 odds ratio, colour), and significance level (-log2 ﻿*Q*-value, size) from a Fisher’s exact test are displayed. j) Associations between copy number signatures and TCGA Asian ethnicity, using TCGA White ethnicity as a reference. k) Associations between copy number signatures and TCGA Black ethnicity, using TCGA White ethnicity as a reference. l) Correlation between copy number signature (*x*-axis) attribution and sex (left), smoking status (middle) and drinking status (right) across TCGA samples. Strength of correlation is indicated by colour (orange=anti-correlated, blue=correlated), ﻿*Q*-value is indicated by size of point. m) Association between copy number signatures (*y*-axis) and median dichotomised age at diagnosis for individual cancer types (*x*-axis). Strength of correlation is indicated by colour (orange=negatively associated, blue=positively associated), *Q*﻿-value is indicated by size of point. Only tumour types/copy number signature combinations with a significant (﻿*Q* < 0.05) association with age at diagnosis are shown.

To identify genetic mechanisms that are potentially causative of copy number signature patterns, we associated somatic cancer-driver gene mutations with copy number signatures and found significant differences between cancer types. A consistent finding across cancer types was a positive association between *TP53* mutations and multiple copy number signatures (Fig. 4a and Supplementary Table 8). *TP53* mutations were also associated with an increased diversity of copy number signatures (Extended Data Fig. 8a; OR = 3.66, *q* = 3.0 × 10^–51^), which provides support for a link between *TP53* alterations and aneuploidy^5^. This result was also confirmed through the observation of CIN signatures such as CN9 in SCS data from RPE1 cells in which *TP53* mutations were induced and from tumours from patients with Li–Fraumeni syndrome (Extended Data Fig. 8b, c and Supplementary Table 8).

Mutations in *RNF43*, *HLA-B*, *HLA-C* and *BRAF* are commonly seen in microsatellite instable colon cancers and were negatively correlated with samples with tetraploid genomes (that is, CN2 attributed; Extended Data Fig. 8d). Microsatellite instability is associated with high immune cell infiltration, whereas aneuploidy is associated with a decrease in leukocyte fraction^50^. Across multiple cancer types, we observed a general trend of decreased leukocyte fractions in cancers with copy number signatures of aneuploidy compared to diploid cancers while accounting for purity (CN1; Extended Data Fig. 8e). Similar to colon cancer, multiple cancer-driver genes were associated with CN1 and CN2 in endometrial cancer, which was largely driven by differential copy number and mutation patterns seen in microsatellite stable and unstable tumours (Extended Data Fig. 8f). Last, we noted a positive association between CN17 and *TP53* mutations in human papilloma virus (HPV) head and neck squamous cell cancer (HNSC) (Extended Data Fig. 8g and Supplementary Table 8). HNSCs are among the most hypoxic of all cancers and are associated with resistance to radiotherapy^49,51^. We therefore reasoned that the association seen here with HRD may actually be driven by hypoxia. Indeed, there was a significant increase in hypoxia scores in HPV-negative HNSC (Extended Data Fig. 8h).

To assess the relationships between copy number signatures and copy number driver genes, we evaluated the associations between attributions of copy number signatures and either homozygous deletions of tumour suppressor genes or amplifications of known proto-oncogenes (Methods). Copy number drivers such as *MDM2*, *EGFR*, *CCNE1*, *MYC* and *ERBB2* were strongly positively associated with the amplicon signatures CN6–CN8 as well as CN17 (Extended Data Fig. 8i and Supplementary Table 8). By contrast, *CDKN2A* was the only homozygously deleted tumour suppressor gene associated with any signature, most commonly CN9.

We also explored the recent links between ancestry and HRD, genomic instability and chromothripsis^52^. The copy number signatures CN17 (HRD), CN6 and CN7 (chromothripsis) and some signatures with unknown aetiology were enriched in tumours of individuals with African ancestry (Extended Data Fig. 8j and Supplementary Table 8). We further associated tumour copy number signatures in people with Asian ancestry and found an enrichment of CN7 (Extended Data Fig. 8k and Supplementary Table 8), a chromothripsis pattern most frequently seen in breast cancers. In contrast to SBS and ID signatures^10^, no associations were found between any copy number signature and cancer risk factors such as sex, smoking status or alcohol consumption (Extended Data Fig. 8l and Supplementary Table 8). Significant associations were found between age and copy number signature attribution in endometrial cancer (Extended Data Fig. 8m and Supplementary Table 8); however, this was driven by subtype differences. That is, serous cancer versus endometrioid endometrial cancer (difference in mean age at diagnosis = 4.7 years, *P* = 8.99 × 10^–5^, two-sided Mann–Whitney test), in which non-endometrioid endometrial cancers are strongly associated with HRD^53^ and enriched in CN17 (OR = 13.6, *P* = 2.5 × 10^–22^, two-sided Fisher’s exact test).

## Discussion

Here we presented a copy number signature framework that provides great utility for the exploration of copy number patterns across multiple cancer types and distinct experimental platforms and exceeds the capabilities provided by mutational signatures of substitutions, IDs or rearrangements. Signatures of substitutions and IDs have translational utility and have been identified across most cancer types and can be generally derived from WGS and, at much lower resolution, WES data^54^. Rearrangement signatures can only be derived exclusively from WGS data and cannot capture important prognostic information such as WGD. By contrast, this copy number signature framework can be applied across all cancer types, which enabled robust and consistent identification of copy number signatures from WGS, WES, reduced representation bisulfite sequencing (RRBS), SCS and SNP6 microarray data.

The identified copy number signatures hold clinical relevance with prognostic implications for patients in which amplicon signatures are observed (Extended Data Fig. 5a). Moreover, the identification of a copy number signature associated with HRD, although not the first such identification^18^, suggests that incorporating such signatures within existing bioinformatics tools for predicting HRD could further increase the accuracy of these tests^55^.

The field of copy number signatures is nascent, with multiple distinct methods previously implemented in distinct tumour types^16–20^. As the field matures, it will become increasingly clear which models are better suited to addressing specific clinical or biological questions. To resolve these questions, pan-cancer analyses that utilize all of these methods will be important, and we present here the first step towards that goal: a mechanism-agnostic pan-cancer compendium of copy number signatures derived from allele-specific profiles.

## Methods

### Utilized datasets

Using SNP6 microarray data, copy number profiles were generated for 9,873 cancers and matching germline DNA of 33 different types from TCGA^6^ using allele-specific copy number analysis of tumours (ASCAT)^56^ with a segmentation penalty of 70 (Supplementary Table 1). In addition, a set of whole-genome sequences from 512 cancers of the International Cancer Genome Consortium that overlapped with tumour profiles in TCGA were analysed^33^ to generate WGS-derived copy number profiles (see below). Last, a set of whole-exome sequences from 282 cancers from TCGA was analysed to generate exome-derived copy number profiles (see below).

### Copy number profile summarization

Copy number segments were classified into three heterozygosity states: heterozygous segments with copy number of (*A* > 0, *B* > 0) (numbers reflect the counts for major allele *A* and minor allele *B*); segments with LOH with copy number of (*A* > 0, *B* = 0); and segments with homozygous deletions (*A* = 0, *B* = 0). Segments were further subclassified into five classes on the basis of the sum of major and minor alleles (TCN; Extended Data Fig. 1e) and were chosen for biological relevance as follows: TCN = 0 (homozygous deletion); TCN = 1 (deletion leading to LOH); TCN = 2 (wild type, including copy-neutral LOH); TCN = 3 or 4 (minor gain); TCN = 5–8 (moderate gain); and TCN ≥ 9 (high-level amplification). Each of the heterozygous and LOH TCN states were then subclassified into five classes on basis of the size of their segments: 0–100 kb, 100 kb–1 Mb, 1 Mb–10 Mb, 10 Mb–40 Mb and >40 Mb (the largest category for homozygous deletions was restricted to >1 Mb). This subclassification was used to capture focal, large-scale and chromosomal-scale copy number changes. In this way, copy number profiles were summarized as counts of 48 combined copy number categories defined by heterozygosity, copy number and size, which we defined as *N* = (*n*_1_,*n*_2_,…,*n*_48_). For a given dataset, the copy number profiles of a set with *S* samples were then summarized as a nonnegative matrix with *S* × 48 dimensions. The segment sizes were selected to ensure that a sufficient proportion of segments were classified in each category, which resulted in a reasonable representation across the pan-cancer TCGA dataset (Extended Data Fig. 1f–h). Two examples, representing a mostly diploid adrenocortical carcinoma (Extended Data Fig. 1i, j) and a copy number aberrant bladder cancer (Extended Data Fig. 1k–l), are provided to illustrate how the segments from a copy number profile are summarized by our framework into a vector of mutually exclusive and exhaustive quantitative features.

### Deciphering signatures of copy number alterations

Copy number signatures were extracted by applying our previously developed approach for creating a reference set of signatures^10^. Specifically, SigProfilerExtractor (v.1.0.17)^21^ was applied to the matrix encompassing all TCGA samples, and separately to each matrix corresponding to an individual tumour type. In brief, SigProfilerExtractor utilizes nonnegative matrix factorization (NMF) to find a set of copy number signatures ranging from 1 to 25 components for each examined matrix. For each number of components, 250 NMF replicates with distinct initializations of the lower dimension matrices were performed on the Poisson resampled data. SigProfilerExtractor was used with default parameters, except for the initializations of the lower dimension matrices, for which random initialization was utilized consistent with our prior analyses of mutational signatures^10,11^. After performing 250 NMFs, SigProfilerExtractor clusters the factorization within each decomposition to automatically identify the optimum number of operative signatures that best explain the data without overfitting these data^21^.

As previously done^10^, the sets of all identified copy number signatures were combined into a reference set of pan-cancer copy number signatures by leveraging hierarchical clustering based on the cosine dissimilarities between each signature. The number of combined signatures is chosen to maximize the minimum average cosine similarity between each signature in a cluster and the mean of all samples in that cluster to ensure that each copy number signature in a cluster has a high similarity to the combined copy number signature for that cluster. Simultaneously, the maximum cosine similarity between mean copy number signatures for each cluster is minimized to ensure that each combined signature is distinct from all others. To avoid reference signatures being linear combinations of two or more other signatures, for each identified signature, a synthetic sample was created with the pattern of the signature multiplied by 1,000 copy number segments. Furthermore, the synthetic sample was resampled with probabilities proportional to the strength of each copy number category in each identified signature. Each resampling was then scanned for activity of all other signatures from the reference set. If a resampled sample can be reconstituted with a cosine similarity >0.95 by 3 or fewer other signatures, the signature used to create the synthetic sample was deemed to be a linear combination of those signatures, and the signature was removed from the global reference set of signatures.

### Reference set of copy number signatures

Initially, 28 pan-cancer copy number signatures were derived from the different SigProfilerExtractor analyses of the 9,873 copy number profiles from SNP microarrays. In silico evaluation and manual curation showed that ten copy number signatures were linear combinations of two or more other signatures. Additionally, three signatures were deemed to be artefactual owing to oversegmentation of copy number profiles. These artefactual signatures were removed from further analyses, as were samples with any attribution of any of these artefactual signatures (116 samples; 1.2% of all TCGA samples). Moreover, samples with >25 Mb of homozygous deletions across the genome were removed from downstream analyses (58 samples), leaving 9,699 samples for full analysis. Following signature assignment (see below), three of the signatures that were removed owing to linear combination were re-extracted within tumour-type-specific assignment (cosine similarity = 1), which indicates that some copy number profiles could not be explained well without these three signatures. As a result, these 3 signatures were reintroduced into the compendium of signatures, leaving a total of 19 signatures. Last, it was observed that a number of samples with high amounts of LOH were poorly explained by the 19 signatures. To remedy this, signatures were extracted from all samples with a proportion of the genome LOH > 0.7. This extraction identified 3 new signatures that were incorporated into the reference set of signatures, giving 22 signatures. One of the newly identified LOH signatures was able to reconstitute 1 of the previous 19 signatures as a linear combination with another signature; therefore the linear combination LOH signature was removed from the reference set, leaving 21 non-artefactual pan-cancer signatures of copy number alteration.

CN1–CN3 form a group of ploidy-associated signatures. CN1 and CN2 display TCNs between 2 and 3–4 respectively, with predominantly >40 Mb heterozygous segments. CN3 consists of predominantly heterozygous segments of TCNs 5–8 with sizes >1 Mb.

CN4–CN8 form a group of amplicon-associated signatures that all have segment sizes predominantly between 100 kb and 10 Mb but with differing TCN or LOH states. CN4 consists of a mixture of LOH segments with a TCN of 1 and heterozygous segments with TCNs 3–4. CN5 consists almost entirely of LOH segments with a TCN of 2. CN6 consists of a mixture of LOH segments with a TCN of 2 and heterozygous segments with TCNs 3–4. CN7 consists of a mixture of heterozygous segments with TCNs of 3–4, 5–8 and 9+. CN8 consists of predominantly heterozygous segments with TCNs of 9+.

CN9–CN12 form a group of signatures with considerable LOH components. CN9 consists of a mixture of LOH segments with a TCN of 2 and heterozygous segments with a TCN of 2, each ranging from 100 kb to 40 Mb, which is suggestive of structural CIN. CN10 consists of a mixture of LOH segments with TCNs 2 and 3–4 and heterozygous segments with TCNs 3–4 between 100 kb and 40 Mb. CN11 consists of a mixture of LOH segments with TCNs 3–4 and heterozygous segments with TCNs 5–8, each at predominantly 1–10 Mb. CN12 consists of mostly LOH segments of a TCN of 2 with sizes >100 kb and additional heterozygous segments of TCNs 3–4 with sizes between 10 and 40 Mb.

CN13–CN16 form a group of signatures with whole-arm-scale or whole-chromosome-scale LOH events, a form of numerical CIN. CN13 is predominantly LOH TCN 1 segments, CN14 is LOH TCN 2 and CN15 is LOH TCN 3–4. CN16 consists of LOH segments with TCNs of 3–4 and heterozygous segments with TCNs of 5–8, each at >40 Mb.

CN17 has been associated with the tandem duplicator phenotype (Fig. 4). This signature consists of LOH segments of TCNs 2 and 3–4 and heterozygous segments of TCNs 3–4 and 5–8, each with segment sizes of 1–40 Mb.

CN18–CN21 originate from unknown processes and are diverse in their copy number patterns. CN18 consists of predominantly heterozygous segments of TCNs 4–8 at >1 Mb, but with appreciable contributions of LOH segments with TCNs 3–4 at >1 Mb and heterozygous segments with TCNs 9+ at >100 kb. CN19 consists of segments between 100 kb and 40 Mb that are heterozygous with TCNs 3–4 or less commonly LOH with a TCN of 1 or 2. CN20 consists of predominantly heterozygous segments with TCNs 3–4 at 100 kb–40 Mb with some heterozygous segments of TCNs 3–4 at 100 kb–10 Mb. CN21 consists of heterozygous segments with a TCN of 2 at >1 Mb and many heterozygous segments with TCNs 3–4 at 100 kb–1 Mb.

### Assignment of copy number signatures to individual cancer samples

The global reference set of copy number signatures was used to assign an activity for each signature to each of the 9,873 examined samples using the decomposition module of SigProfilerExtractor^21^. For the assignment, the information of the de novo signature and their activities assigned to each sample were used to implement the decomposition module with default parameters, except for the NNLS addition penalty (nnls_add_penalty), which was set to 0.1, the NNLS removal penalty (nnls_remove_penalty), which was set to 0.01, and the initial removal penalty (initial_remove_penalty), which was set to 0.05. Signatures were assigned to samples in both tumour-specific evaluations and in a pan-cancer evaluation. As previously done^10^, the signature attributions from either tumour-specific or pan-cancer evaluations that gave the best cosine similarity between the input sample vector and the reconstructed sample vector were used as the attributions for that sample in all subsequent analyses.

### Copy number signatures derived from WGS and WES data

A set of samples from TCGA with both SNP array and exome sequencing data were selected (*n* = 282). Copy number profiles were generated from the exome sequencing data using ASCAT across all of the dbSNP common SNP positions with a segmentation penalty ranging from 20 to 140. Signatures were re-extracted for these 282 samples from both the SNP-array-derived copy number profiles and the exome-derived copy number profiles, and the resulting signatures were compared.

For WGS data, we examined 512 whole-genome sequenced samples from the PCAWG project overlapping with TCGA samples with microarray data. Copy number profiles from WGS data were generated using ASCAT across the SNP6 positions, with a segmentation penalty ranging from 20 to 120. Signatures were extracted for samples with both SNP6-microarray-derived copy number profiles and the WGS-derived copy number profiles, and the extracted signatures were compared. In all cases, a segmentation penalty of 70 gave the best concordance for both copy number profiles and extracted copy number signatures based on SNP6 microarray, WGS and WES data.

### Copy number signatures derived from different copy number callers

A set of 3,175 allele-specific copy number profiles called using the ABSOLUTE^57^ algorithm were obtained. Copy number signatures were extracted from the 3,175 ABSOLUTE profiles, as well as re-extracted for the 3,175 corresponding ASCAT profiles. Signatures were compared using cosine similarity with between 2 and 12 signatures extracted, and with the sigProfiler suggested solution of 4 signatures extracted.

### Mapping copy number signatures to the landscapes of cancer genomes

See Supplementary Methods for details of mapping copy number signatures back onto the reference genome.

For all mapping analyses, *P* values were adjusted for multiple testing as appropriate for Monte Carlo testing^58^.

### Associations between copy number signatures and events defined by genomic region

Localized events (chromothripsis^33^ and amplicon structure^30^) identified using WGS data were associated with mapped copy number signatures from TCGA for all available matching samples (chromothripsis *n* = 657; amplicon *n* = 1,703). Each segment in every sample was categorized as overlapping or non-overlapping of a localized event. For each copy number signature, the association was then tested using two-sided Fisher’s exact test on a contingency table of segments categorized as overlapping or non-overlapping of a localized event and assigned to or not assigned to the given copy number signature across all samples. Multiple-testing correction was performed using the Benjamini–Hochberg method.

### Genome-doubled copy number signatures

With the copy number categories being defined as 0, 1, 2, 3–4, 5–8 and 9+, it is possible to artificially ‘genome double’ any copy number category, other than 0, by assigning it to the next highest copy number category. In this way, we artificially ‘genome doubled’ each signature by assigning the count for each copy number class to its next highest copy number class. First, the copy number 1 class is assigned a count of 0, then each copy number class is assigned the count of the preceding copy number class. For example, copy number class of 2 is assigned to the previous copy number class of 1, 3–4 assigned previous 2, and so on, until finally the copy number 9+ class is assigned a count that is the sum of the previous copy number 5–8 class and 9+ class. During this conversion, LOH and size categories were retained so that the only shift is in copy number. Having performed this conversion, cosine similarities between the artificially genome-doubled signatures and the original signatures were calculated. Any genome-doubled and original signature pair that had a cosine similarity of >0.85 was considered to contain a pair of signatures with analogous copy number patterns distinguished only by their genome-doubling status.

### Associations between copy number signatures and ploidy

Ploidy for each copy number profile was calculated as the relative length weighted sum of TCN across a sample. The proportions of the genome that displayed LOH (pLOH) were also calculated. Samples with a ploidy above −3/2 × pLOH + 3, meaning an LOH-adjusted ploidy of 3 or greater, were deemed to be genome-doubled samples. By contrast, samples with a ploidy above −5/2 × pLOH + 5, meaning an LOH-adjusted ploidy of 5 or greater, were deemed to be twice genome-doubled samples. All other samples were considered as non-genome-doubled samples. Each signature (CN1–CN21) was associated with each genome doubling category (GD×0, GD×1 and GD×2) using one-sided Fisher’s exact test on a contingency table with samples categorized by whether the samples have >0.05 attribution to the given copy number signature or not, and whether the sample has the given genome doubled category or not. All *P* values were corrected for multiple hypothesis testing using the Benjamini–Hochberg method.

### Associations between copy number signatures and known cancer risk factors

Associations between attributions of copy number signatures and attributions of SBSs, IDs and doublet-base signature exposures^10^ were performed using Kendall’s rank correlation. Only the significant associations found in both cancer-type-specific and pan-cancer analysis are reported. For the cancer risk association analyses, copy number signatures were associated with sex^59^, tobacco smoking^60^ and alcohol drinking status^61^. For each copy number signature, the association was conducted using two-sided Fisher’s exact test on a contingency table of a clinical feature categorized as present or absent and assigned to or not assigned to the given copy number signature across all samples. All *P* values were corrected for multiple hypothesis testing using the Benjamini–Hochberg method.

Associations between copy number signature attribution (binarized to present or absent) and the TDP (also binarized to present or absent)^29^ were performed using two-sided Fisher’s exact test (*n* = 882). This was performed for each copy number signature separately. All *P* values were corrected for multiple hypothesis testing using the Benjamini–Hochberg method, and only associations with *q* < 0.05 are reported.

Associations between copy number signature attribution (binarized to present or absent) and driver-gene single nucleotide variant (SNV) and ID mutation status^40^ were performed within tumour types using two-sided Fisher’s exact test (*n* = 6,543 across all cancer types). This was performed for all copy number signature/gene combinations for which the gene was mutated in the given cancer type and the copy number signature was observed in the given cancer type. All *P* values were corrected for multiple hypothesis testing using the Benjamini–Hochberg method, and only associations with both *q* < 0.05 and |log_2_(OR)|>1 are reported.

Driver copy number alterations of COSMIC cancer gene census genes^62^ were defined as follows: (1) homozygous deletion (CN = (0, 0)) of genes listed as deleted (D) in COSMIC mutation types; or (2) amplification (CN > 2 × ploidy + 1) of genes listed as amplified (A) in COSMIC mutation types. Associations were then performed on copy number driver alterations for SNV and ID driver gene alterations as outlined above (*n* = 9,699 across all cancer types).

The diversity of copy number signatures, as defined by Shannon’s diversity index, was associated with both SNV and ID and copy number driver gene mutations using a logistic regression model with binary diversity (>0, =0) as the dependent variable, and tumour type and gene mutation status as independent variables. LGG was taken as the reference tumour type. Only driver genes with >250 mutant samples in the dataset were included in the model.

Associations between copy number signature attribution (binarized to present or absent) and age at diagnosis (binarized to above or below median separately for each cancer type) were performed within cancer types using two-sided Fisher’s exact test (*n* = 8,841 across all cancer types). All *P* values were corrected for multiple hypothesis testing using the Benjamini–Hochberg method, and only associations with both *q* < 0.05 and |log_2_(OR)|>1 are reported.

Leukocyte counts were obtained from TCGA^50^. The leukocyte fraction was associated with copy number signatures using a logistic regression model with binarized leukocyte fraction (fraction > or ≤ median fraction) as the dependent variable, and binarized copy number signature attribution (0, >0 attribution) and ASCAT estimated tumour purity as independent variables. All *P* values were corrected for multiple hypothesis testing using the Benjamini–Hochberg method.

### Copy number signatures and defective HR

Signatures were tested for enrichment in tumour types using one-sided Mann–Whitney tests of signature attribution in a given tumour type versus all other tumour types. This was performed for all signature and tumour combinations. All *P* values were corrected for multiple hypothesis testing using the Benjamini–Hochberg method.

The following core HR repair pathway member genes were chosen for interrogation: *BRCA1*, *BRCA2*, *RAD51C* and *PALB2* (refs. ^63,64^). Copy number alterations across these genes were identified based on ASCAT copy number profiles for homozygous deletions (that is, CN = (0, 0)) and LOH (that is, CN = (>0, 0)). Somatic SNVs and IDs were taken from ref. ^40^. Pathogenic germline variants in *BRCA1* and *BRCA2* were taken from ref. ^65^. Samples were deemed as bi-allelically mutated for the HR pathway if homozygously deleted or if more than one of any of the other classes of alteration were present within any of the HR pathway genes. Mono-allelic loss was defined as one of any of the non-homozygously deleted alterations within any of the HR pathway genes. Wild type was defined as no alterations in any HR pathway genes. The associations between HR pathway status and CN17 were then restricted to only breast (*n* = 589), ovarian (*n* = 309) and pan-cancer (*n* = 4,919). Two-sided Fisher’s exact tests were performed between wild-type and mono-allelic samples, between wild-type and bi-allelic samples, and between mono-allelic and bi-allelic HR pathway status samples. All *P* values were corrected for multiple hypothesis testing using the Benjamini–Hochberg method.

A further multivariate logistic regression model was utilized with CN17 attribution (>0 or 0) as the dependent variable, and *BRCA1*, *BRCA2*, *RAD51C*, *PALB2*, *FBXW7*, *CDK12* mutational status, categorized as wild type, mono-allelic or bi-allelic as previously described, as independent variables, to test associations between the mutation status of individual HR pathway genes and CN17.

Orthologous scores of HRD were calculated using scarHRD^61^. Associations between scarHRD scores and CN17 were tested using two-sided Fisher’s exact tests, with CN17 categorized as present or absent, and scarHRD scores categorized as positive or negative around thresholds of both 42 (which has been described as an adequate threshold in breast cancer^61^) and 63 (which has been described as an adequate threshold in ovarian cancer^66^). Furthermore, we associated the presence or absence of CN17 with continuous scarHRD scores using two-sided Mann–Whitney test.

To test associations between promoter hypermethylation of the HR machinery and CN17, TCGA methylation *β* values were downloaded from https://portal.gdc.cancer.gov/ and TCGA-normalized gene expression RSEM values were downloaded from https://gdac.broadinstitute.org/

Relationships between log_10_(RSEM) values and mean TSS200 and TSS1500 associated methylation probe *β* values were initially inspected in breast cancer to determine a threshold mean *β* value for determining promoter hypermethylation and subsequent epigenetic silencing of *BRCA1*. This threshold was set at mean *β* > 0.7.

CN17 attribution was associated between *BRCA1* promoter hypermethylated breast cancer samples and both genomic *BRCA1* wild-type and bi-allelically mutated breast cancer samples using two-sided Mann–Whitney test. This analysis was extended to a pan-cancer association, performing two-sided Fisher’s exact tests between signature attribution or not, and promoter hypermethylation (mean TSS200 and TSS1500 *β* > 0.7) or hypomethylation (mean TSS200 and TSS1500 *β* ≤ 0.7). *P* values were corrected for multiple testing using the Benjamini–Hochberg method.

### Copy number signatures associated with hypoxia

Gene-expression-derived scores of hypoxia from 8,006 TCGA tumours were used^49,67^. A linear regression with hypoxia score as the dependent variable, and binarized copy number signature attributions (>0, =0) as well as tumour type as independent variables.

### Copy number signatures associated with complex rearrangements

Assignment of rearrangement phenomena to PCAWG samples were used^31^. Associations of each re-arrangement phenomenon with each copy number signature were evaluated using two-sided Fisher’s exact tests of copy number signature non-attributed or attributed (=0, >0) against rearrangement phenomenon presence or absence. *P* values were corrected for multiple testing using the Benjamini–Hochberg method.

### Copy number signatures associated with HPV in HNSC

We used HPV testing status from TCGA HNSCs obtained from ref. ^68^. HPV status was associated with copy number signature attribution using two-sided Fisher’s test. *P* values were corrected for multiple testing using the Benjamini–Hochberg method. Furthermore, hypoxia scores (see above) were associated with HPV status using two-sided Mann–Whitney test.

### Copy number signature associated with ethnicity

Ethnicity information for 11,160 individuals from TCGA was taken from the TCGA Clinical Data Resource^59^. Copy number signatures (binarized to present/absent) were associated between Black/White ethnicity and between Asian/White ethnicity separately using two-sided Fisher’s exact tests. *P* values were corrected for multiple testing using the Benjamini–Hochberg method.

### Copy number signatures associated with changes of overall survival

Survival data for 11,160 individuals from TCGA were obtained from the TCGA Clinical Data Resource^59^. Univariate disease-specific survival analysis for signatures was performed using a log-rank test and Kaplan–Meier curves in R, with groups being unattributed (attribution = 0) and attributed (attribution > 0) for each signature separately, or for summed attributions of a set of signatures (for example, amplicon signatures).

Multivariate disease-specific survival analysis was performed using the Cox’s proportional hazards model in R with Boolean attributed/non-attributed variables for each copy number signature and tumour type as covariates. To account for potential violations of Cox’s model’s proportional hazards assumption, we also conducted the same analysis using the accelerated failure time model with the Weibull distribution using the flexsurvreg function in R. All *P* values were corrected for multiple hypothesis testing using the Benjamini–Hochberg method.

### Simulating copy number profiles

See Supplementary Methods for details of the methods used to simulate copy number profiles from various processes.

### Single-cell isolation, FACS analysis and DNA library generation for USARC ploidy estimation

Fresh frozen tumour tissue was thawed on ice, dissected and homogenized with 500 µl of lysis buffer (NUC201-1KT, Sigma). Following the release of single nuclei, samples were centrifuged, and the resulting precipitate removed. A 10 µl sample was taken to count and evaluate the extracted nuclei. The lysate was cleaned using a sucrose gradient following the manufacturer’s instructions (NUC201-1KT, Sigma). After cleaning, the nuclei were centrifuged at 800*g* for 5–10 min at 4 °C and resuspended in PBS, supplemented with 140 µg ml^–1^ RNase (19101 Qiagen) and stained with 1 µg ml^–1^ DAPI (Sigma-Aldrich), and 2.5 µg ml^–1^ Ki-67 antibody (BioLegend) per 1 million cells in 100 µl. Stained nuclei were analysed using a FACS Aria Fusion cell sorter (BD bioscience) and FACS DIVA software (v.8.0.1). Cells were sorted using a 130-μm nozzle with 12 psi set for sheath pressure. Each gated population of interest was collected into a separate 1.5-ml tube, and a custom sort precision of 0-16-0 (Yield-Purity-Phase) was used. For cells collected into plates, the sort precision used was Purity, defined as 32-32-0 (Yield-Purity-Phase). DAPI was measured using a 355-nm UV laser with a 450/50 bandpass filter. Ki-67 was measured using a 635-nm red laser with a 670/30 bandpass filter. Forward scatter and side scatter were both measured from a 488-nm blue laser on a linear scale. DAPI was also measured on a linear scale and was used to estimate the DNA content per single cell. A control diploid cell line was used to establish accurate ploidy measurements before sorting. Forward versus side scatter area was used to exclude debris, whereas the height versus area of the DAPI fluorescence was used to exclude doublets. FACS analysis revealed the presence of three major aberrant cell populations (Supplementary Methods), including a haploid population (1*n*), a nearly diploid population (2*n*, Ki-67 positive) and a WGD population (3*n+*). A non-proliferating, non-aberrant, normal cell population was also identified (2*n*, Ki-67 negative).

Once sorted, single nuclei suspensions were processed using a Chromium Single Cell DNA Library & Gel Bead kit (10X Genomics, PN-1000040) according to the manufacturer’s instructions, with a target capture of 1,000–2,000 cells. The resulting barcoded single-cell DNA libraries were sequenced with an Illumina HiSeq 4000 system using 150 bp paired-end sequencing with a coverage ranging from 0.01 to 0.08 X per cell. Germline bulk WGS was also performed on a XTen instrument (Illumina) as previously described^16^. Copy number signatures were also evaluated in single cells harbouring chromothripsis, as well as WGD events using sequencing data that had already been generated from a cell-based model system linking chromothripsis and hyperploidy^69^.

### Single-cell allele-specific copy number alteration calling using ASCAT.sc

USARC single-cell paired-end reads generated using the chromium single cell CNV platform were processed using the 10X Genomics Cell Ranger DNA Pipelines (https://support.10xgenomics.com/single-cell-dna/software/pipelines/latest/what-is-cell-ranger-dna). Following sample demultiplexing, data were aligned to the GRCh38 reference genome and a barcoded BAM file was obtained for every considered single cell per individual USARC ploidy population. To analyse each barcoded BAM file and derive total copy number alterations for each single cell, we then applied ASCAT.sc v.1.0 (https://github.com/VanLoo-lab/ascat), our in-house pipeline, to analyse single-cell and shallow coverage WGS data. Similar to its predecessor ASCAT, which measures allele-specific copy number alterations in bulk tumour data^56^, ASCAT.sc infers single-cell TCN states from changes in the relative read depth (logR). Importantly, ASCAT.sc derives the logR from the number of reads aligning in different genomic bins, unlike ASCAT, which relies on both the logR and the allelic imbalance (otherwise known as the B-allele frequency) at SNP loci identified as heterozygous in the germline. Thus, ASCAT.sc utilizes logR shifts to segment the genome into regions with constant TCN states, thereby assigning integer copy number profiles to single cells. For single-cell allele-specific copy number alterations, we first performed single-cell segmentation using multiple piecewise constant fitting^70^ using the R package copynumber v.1.26.0 (https://bioconductor.org/packages/release/bioc/html/copynumber.html). We then provide ASCAT.sc with the available matched-normal germline sample and generate phased germline SNPs using Beagle (v.5.1)^71^ as part of the subclonal copy number calling pipeline, Battenberg^72^. ASCAT.sc then uses single cell logR values alongside phased SNP data, as well as allele counts for heterozygous SNPs (generated using alleleCount; https://github.com/cancerit/alleleCount) to calculate allele-specific copy number alterations in single cells. These results can be used to group cells into distinct tumour subclones while also excluding noisy single cells.

### Copy number signatures on single-cell copy number profiles

For all single-cell datasets, adjacent genomic bins within a chromosome with the same major and minor copy number were combined into a single segment. Genomic bins for which no copy number state was assigned were removed from the profiles. Copy number summaries were then generated, and TCGA copy number signatures were scanned using sigProfilerSingleSample on all cells.

Because of the nature of the undifferentiated sarcoma for which single-cell sequencing was performed (near-genome-wide LOH), the majority of the genome should be LOH for tumour cells, and a minority of the genome should be LOH for normal cells. However, we observed a number of cells for which the majority of the genome had a copy number of (1, 4). This is an erroneous copy number pattern, which occurred owing to the difficulty of calling LOH from single-cell data in the context of multiple genome-doubling events. Cells with a proportion of the genome LOH < 0.4 and a proportion of the genome with imbalanced copy number (major CN!=minorCN) > 0.6 were excluded from further analysis to remove erroneous profiles.

For an assessment of copy number signatures in genomically unstable single cells, BAM files from *TP53* mutant RPE1 cells were downloaded^69^. Copy number profiles were generated as for the USARC single cell data, and scanned for signatures using sigProfilerSingleSample.

### FACS and copy number profiling of ploidy populations for RRBS

The sorting strategy for RRBS workflows was modified to collect groups of cells belonging to different ploidy populations based on DAPI staining (Supplementary Methods). Five tumour samples were processed in this manner, DNA was extracted using a Quick-DNA Miniprep Plus kit (Zymo, D4068) and library preparation and quality control was performed using an Ovation RRBS Methyl-Seq system (Nugen, 0353, 0553) according to the manufacturer’s instructions. Paired-end sequencing was performed on an Illumina NovaSeq instrument using an S1 flowcell 100 cycles (single end). Allele-specific copy number calling was performed using CAMDAC (https://github.com/VanLoo-lab/CAMDAC).

Copy number signatures for the 4 ploidy-sorted populations and the bulk population were extracted using sigProfilerExtractor, setting the number of signatures to extract at 4. Artificial genome-doubling of the identified signatures was performed as described above. The 5 samples were also scanned for the 21 TCGA signatures using sigProfilerSingleSample; identified copy number signatures were categorized by their predominant genome-doubling association (see above), and the prevalence of individual genome doubling category (WGD×0, WGD×1, WGD×2) signatures was evaluated.

### Copy number signatures in germline *TP53* mutant cancers

We used Battenberg-derived^72^ copy number profiles of WGS data from cancer samples of patients with Li–Fraumeni disease^73,74^. Additional clinical metadata and highly curated sequencing data for additional cases were obtained from D.M., A.S. and N.L.

### Data analysis

All signatures decompositions, assignments and matrix generations were performed using the sigProfiler suite (see above) of Python packages using Python v.3.7.1.

All statistical analyses were performed in R v.4.0.2. Plotting was performed with base R or with packages ggplot2, ggrepel, RColorBrewer, circlize, ComplexHeatmap, colorspace, seriation, dendextend, beanplot and corrplot. Survival analysis was performed with the R packages survival and survminer. Multiple testing correction was performed using qvalue. Cosine similarities were calculated using the cosine function from lsa. TSNE analysis was performed using Rtsne. Data handling was performed with GenomicRanges, tidyr, stringr, parallel and gtools.

### Ethics

Informed consent from patients and ethical approval for tissue biobanking was obtained through the UCL/UCLH Biobank for Studying Health and Disease (REC reference: 20/YH/0088; NHS Health Research Authority). Approval for the study and ethics oversight was granted by the NHS Health Research Authority (REC reference: 16/NW/0769).

### Reporting summary

Further information on research design is available in the Nature Research Reporting Summary linked to this paper.

## Online content

Any methods, additional references, Nature Research reporting summaries, source data, extended data, supplementary information, acknowledgements, peer review information; details of author contributions and competing interests; and statements of data and code availability are available at 10.1038/s41586-022-04738-6.

## Supplementary information

Supplementary Information This file contains the Supplementary Methods and Supplementary Figs. 1 and 2. The methods include a description of the protocol for mapping copy number signatures to the reference genome and simulating copy number profiles from assumed processes. Supplementary Figs. 1 and 2 display the FACS gating strategies for ploidy sorting of tumour cells, with associated descriptions of the methods used.

Reporting Summary

Supplementary Table 1 ASCAT copy number profiles and copy number summary vectors for SNP6, WGS and WES data. Includes abbreviations for TCGA cancer types.

Supplementary Table 2 Identified copy number signature definitions and their attributions across TCGA.

Supplementary Table 3 Ploidy-association data and results, including copy number profiles and TCGA signature attributions from RRBS sequencing of a ploidy-sorted sarcoma, association results with genome doubling and ploidy.

Supplementary Table 4 Median attributions and proportion of samples attributed to each signature for each cancer type in TCGA.

Supplementary Table 5 Chromothripsis-related association results, survival-association results and mapping recurrence of CN8.

Supplementary Table 6 fLOH and cLOH signature mapping recurrence and input copy number profiles, copy number summaries and TCGA copy number signature attributions for single-cell sequencing data.

Supplementary Table 7 Associations related to HR deficiency, including associations with SBS/indel signatures and hypoxia.

Supplementary Table 8 Associations with driver gene alterations, Li–Fraumeni data inputs and outputs, RPE1 *TP53* mutant cell line data inputs and outputs.

## Data Availability

ASCAT copy number profiles can be found at https://github.com/VanLoo-lab/ascat/tree/master/ReleasedData/TCGA_SNP6_hg19 Data for single-cell sequencing (EGAS00001006144) and RRBS sequencing (EGAS00001006143) are deposited in the European Genome-Phenome Archive.

## Code availability

Code for summarizing copy number profiles into 48-length vectors can be found at https://github.com/AlexandrovLab/SigProfilerMatrixGenerator.

Code for extracting copy number signature can be found at https://github.com/AlexandrovLab/SigProfilerExtractor.

Code for decomposing copy number summaries into known copy number signatures can be found at https://github.com/AlexandrovLab/SigProfilerSingleSample.

Code for artificially genome-doubling signatures, mapping signatures to the genome, assessing signature recurrence, simulating copy number profiles and bespoke scripts can be found at https://github.com/UCL-Research-Department-of-Pathology/panConusig.
