# Supplementary material for: Signatures of copy number alterations in human cancer
Source: Nature. 2022 Jun 15;606(7916):984–91. doi: 10.1038/s41586-022-04738-6 (PMC9242861; doi:10.1038/s41586-022-04738-6)
Supplement: Supplementary file 1 — This file contains the Supplementary Methods and Supplementary Figs. 1 and 2. The methods include a description of the protocol for mapping copy number signatures to the reference genome and simulating copy number profiles from assumed processes. Supplementary Figs. 1 and 2 display the FACS gating strategies for ploidy sorting of tumour cells, with associated descriptions of the methods used. [file 41586_2022_4738_MOESM1_ESM.docx]

# Supplementary Methods

### **Mapping copy number signatures to the landscapes of cancer genomes**

Given the original copy number profiles, the identified signature matrix of $c$ copy number classes by $f$ signatures, and the signature activity matrix of $s$ samples by $f$ signatures, it is then possible to map signatures to the genomic landscape for each cancer sample. The probability of each copy number class, ***c***, having originated from each signature, ***i*** from a total of ***I*** signatures, in a sample ***j*** can be defined as:

$m_{i,j,c}=\frac{\boldsymbol{f}_{c,i}\boldsymbol{e}_{i,j}\boldsymbol{l}_{j}}{\sum_{k=1}^{I} \boldsymbol{f}_{c,k}\boldsymbol{e}_{k,j}\boldsymbol{l}_{j}}$,

where $\boldsymbol{f}$ is the normalised signature matrix, $\boldsymbol{e}$ is the normalized attribution matrix, and $\boldsymbol{l}$ is a matrix of the number of segments in the copy number profile of each sample. The likelihood of each signature contributing to a given genomic window, here taken as each chromosome, is then the sum of copy number class probabilities for each segment in that window:

$$p_{i,j,w}=\sum_{x=1}^{l_{j,w}} m_{i,j,c_{x}}$$

Once these chromosome likelihoods have been calculated, the individual segments in a chromosome are assigned to their maximum likelihood signature. Once copy number signatures have been mapped to the genome at a segment level, it is possible to interrogate the recurrence of signatures across the genome for a given set of copy number profiles. To do this, the genome is binned into 1Mb tiled windows. Within each window, the number of samples with a segment of a given copy number signature that overlaps the window is computed. This is repeated for each signature in each window.

Enrichment of copy number signatures in regions was evaluated using a bespoke bootstrapping approach. For a given signature (e.g. CN8) all copy number profiles across TCGA that were attributed that signature were used as a pool to draw copy number information from. For each chromosome [1…22,X] a random sample is chosen to assign the CN breakpoints. The copy number signature assigned to each CN segment in the chromosome is then drawn from the copy number signatures of all segments across the pool of samples that match the segment size class of the CN segment in question. This is repeated for *n* samples, where *n* matches the number of samples in the dataset that recurrence was calculated for e.g. 56 GBM samples subject to CN8. The recurrence of each signature in each genomic bin in this randomly drawn set of copy number profiles is calculated. This process is repeated for 10,000 simulated datasets. The p-value at each genomic bin is then calculated as the proportion of simulated datasets in which the recurrence of the given signature is greater than the recurrence in the observed dataset. P-values are adjusted for multiple testing as appropriate for Monte Carlo testing^1^.

**Simulating copy number profiles**

*Simulation framework:* Genomes were initialized as 23 pairs of individual chromosomes, with lengths corresponding to those seen in the human genome, where the 23^rd^ pair could be either *X, X* or *X, Y*. Each chromosome was initialized as a data table with chromosome (1-22, X, Y), start position, end position, and allele (either A or B). Genomic events were recorded as altering one of these data tables in the appropriate way, adding or removing segments as necessary. Gains and losses: The log_10_(size) of sub-chromosomal gains were drawn from a Gaussian mixture with components:

**N**(μ=5.961351, σ^2^=0.4199448),

**N**(μ=7.786183, σ^2^=0.1068539),

at proportions p_1_=0.7360366 and p_2_=1-p_1_. The log_10_(size) of sub-chromosomal losses were drawn from a gaussian mixture with components:

**N**(μ=6.188331, σ^2^= 0.5686788),

**N**(μ=7.588125, σ^2^= 0.1326166),

at proportions p_1_=0.6472512 and p_2_=1-p_1_. The parameters for the various distributions were estimated from samples in TCGA that were predominantly diploid (CN1+CN9 attribution>0.8) from segments that were copy number 1 for the loss distributions, and copy number 3 for the gain distributions. Parameters were estimated using a Gaussian mixture model on the log_10_(sizes) of the appropriate segments with two components due to the bimodal nature of the segment length distributions.

First the chromosome on which the gain/loss will occur is randomly sampled with probabilities 1/*n*, where *n* is the number of separate chromosomes in the current genome. The event size, λ ,is then drawn from the previously stated multinormal distributions; if an event size greater than the chromosomal size is drawn, then a new size is drawn. The start of the event, *b_1_*, is then drawn from a uniform distribution,

*b_1_*~**U**(1,*e-*λ),

where *e* is the cumulative length of the chosen chromosome, and the end of the event, *b_2_*=*b_1_*+λ.

Gains are treated as tandem duplications, so that the gained region is inserted immediately after the start breakpoint. On unaltered chromosome, this will alter the chromosome from a single segment with start=1 and end=*e* to a chromosome with four segments, with starts=[1,*b_1_+1*,*b_1_+1*,*b_2_+1*] and ends=[*b_1_*,*b_2_*,*b_2_*,*e*], each with the chosen chromosome identity and allele; note that this will eventually lead to a copy number profile with 3 segments with starts==[1,*b_1_+1*,*b_2_+1*] and ends=[*b_1_*,*b_2_*,*e*]. A loss will instead lead to a chromosome with two segments with starts=[1,*b_2_*] and ends=[*b_1_*,*e*].

#### Simulating chromothripsis: For chromothriptic events, the log_10_(number of segments) for the resulting chromosome is drawn from a normal distribution:

*n*~**N**(μ=1.3, σ=0.3),

while the log_10_(length) of segments are drawn from a normal distribution

λ~**N**(μ=6, σ=0.7),

and the start of the chromothriptic event is drawn from a uniform distribution:

**U**(1,*e*-$\sum_{1}^{n} l_{n}$),

where *e* is the size of the chromosome. The parameters for the distributions were chosen to match the empirical distributions observed in TCGA chromosomes that were called as chromothriptic in the PCAWG dataset.

The breakpoints of the chromothriptic event, [*b_1_*,…,*b_n-1_*], are then the cumulative sums of the segment sizes, apart from the first breakpoint which is 1. The chromosome is then broken into *n* segments by their cumulative lengths, defined by the breakpoints. Whether to lose a segment is drawn from a binomial distribution:

δ_x_~**Binom**(1,0.5).

All segments were removed where δ_x_=1. The remaining segments were then randomly reversed if:

ρ_x_~**Binom**(1,0.5)=1.

Lastly, the remaining segments were resampled without replacement so that their order is randomized, and are then concatenated together. The chromothriptic chromosome replaces the original chromosome that it originates from.

#### Genome doubling and chromosomal gains/losses: All chromosomes in the set of chromosomes are duplicated to simulate genome doubling. For chromosomal gains, a single chromosome is duplicated, whereas for chromosomal losses a single chromosome is removed.

#### Calculating copy number: Once an assortment of chromosomes has been simulated from a mixture of the previously described processes, the combined copy number across all derivative chromosomes must be calculated across the reference genome. For each reference chromosome, x, all segments across the derivative chromosomes that derive from x are collated, and the breakpoints across x are defined as the ordered unique set of start or end positions of those segments. Then the copy number for segment i_x_, is calculated for each allele separately; the A allele copy number is the count of A allele segments in all derivative chromosomes that overlap the segment defined between b_i,x_ and b_i+1,x_, and similar for the B allele copy number. Combined across all reference chromosomes, this gives an allele-specific copy number profile.

#### Combinations of simulations: The following simulations were performed, for 100 samples each:

- CINx10 – 10 random gain or loss events.
- CINx50 – 50 random gain or loss events.
- CINx10->WGD – 10 random gain or loss events, followed by WGD.
- CINx50->WGD – 50 random gain or loss events, followed by WGD.
- CINx5->WGD->CINx50 - 5 random gain or loss events, followed by WGD, followed by 50 random gain or loss events.
- CINx5->WGD->CINx25->WGD->CINx25 - 5 random gain or loss events, followed by WGD, followed by 25 random gain or loss events, followed by WGD, followed by 25 random gain or loss events.
- Chromo. – Chromothripsis of a random chromosome.
- Chromo.->WGD – Chromothripsis of a random chromosome, followed by WGD.
- Chromo.->Amp. – Chromothripsis of a random chromosome, followed by chromosomal gain of the derivative chromothriptic chromosome.
- Chromo.->Amp.->WGD - Chromothripsis of a random chromosome, followed by chromosomal gain of the derivative chromothriptic chromosome, followed by WGD.
- Chromo.->Amp.x5->WGD. Chromothripsis of a random chromosome, followed by chromosomal gain of the derivative chromothriptic chromosome five times, followed by WGD.

For random gain/loss events, a binomial draw was used to decide whether a gain or loss occurred, with p_gain_=0.4.

## Supplementary Figure 1


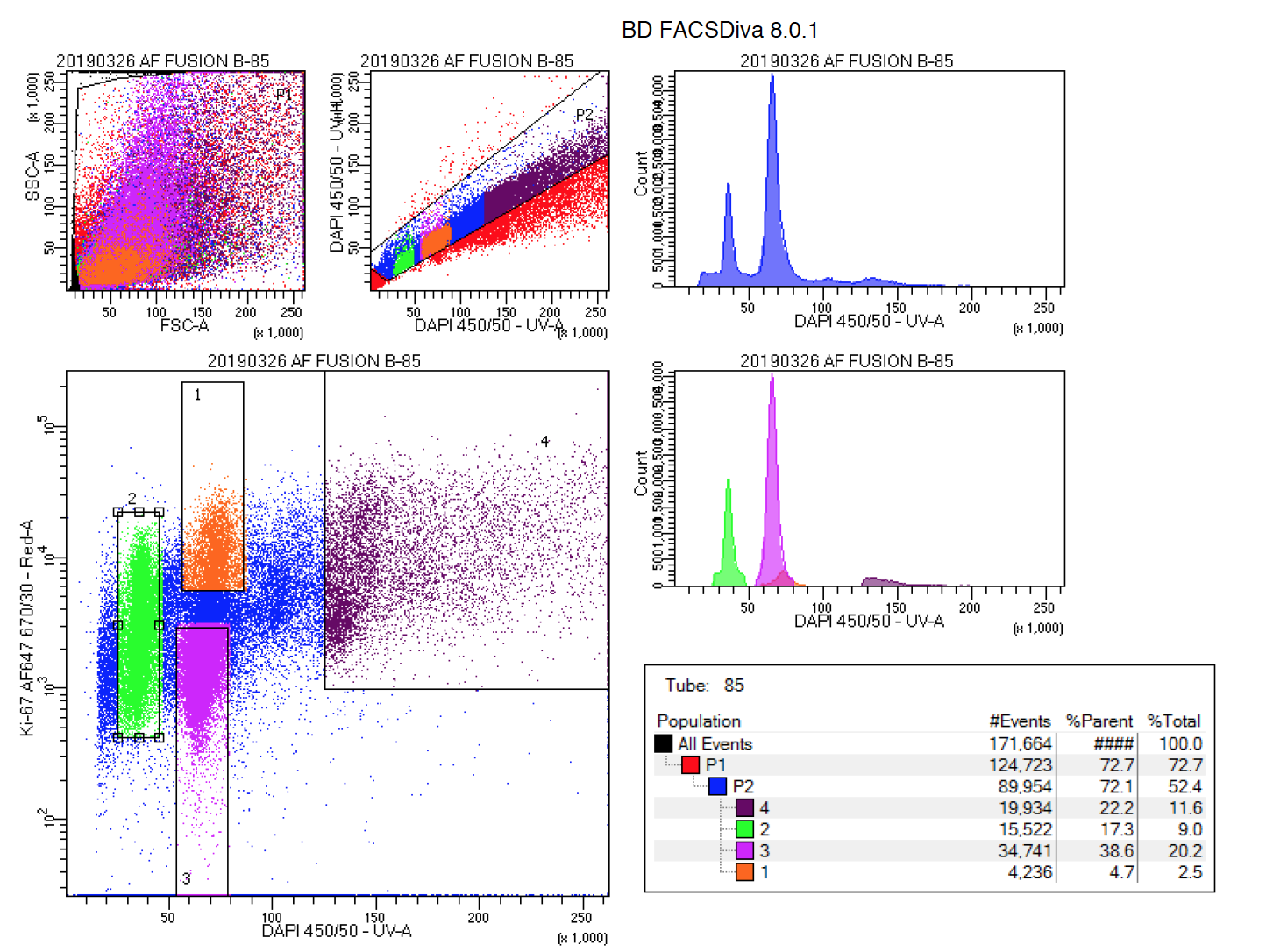


Gating strategy for FACS ploidy sorting of an undifferentiated sarcoma for downstream single cell sequencing. DAPI was measured using a 355 nm UV laser with a 450/50 bandpass filter. Ki-67 was measured using a 635 nm Red laser with a 670/30 bandpass filter. Forward scatter and side scatter were both measured from a 488nm blue laser on a linear scale. DAPI was also measured on a linear scale and was used to estimate DNA content per single cell. A control diploid cell line was used to establish accurate ploidy measurements prior to sorting. Forward vs. side scatter area was used to exclude debris, while the height vs area of the DAPI fluorescence was used to exclude doublets. FACS analysis revealed the presence of three major aberrant cell populations within our USARC, including a haploid population (1n), a nearly diploid population (2n, Ki-67 positive) and a WGD population (3n+). A non-proliferating, non-aberrant, normal cell population was also identified (2n, Ki-67 negative).

## Supplementary Figure 2


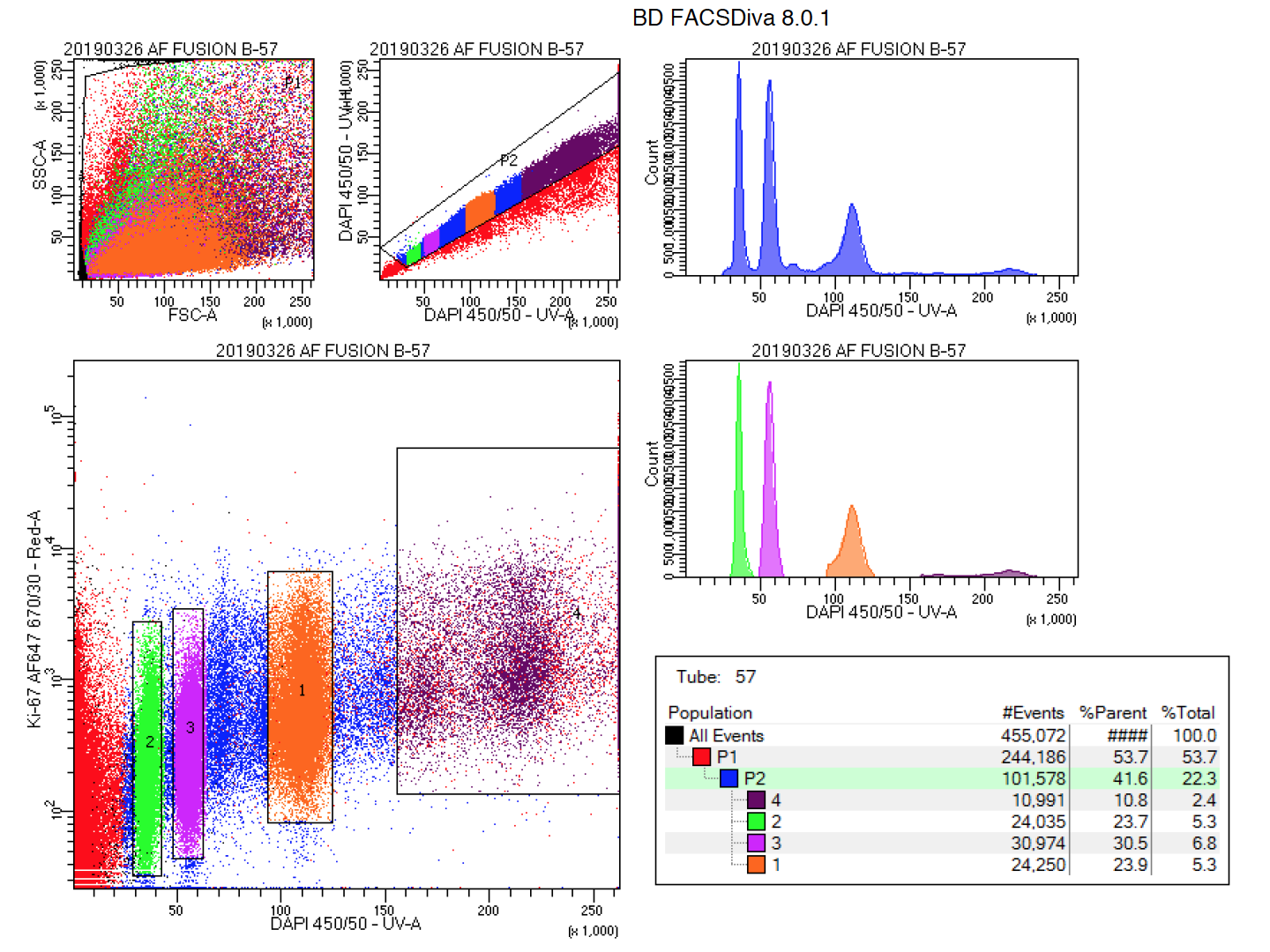


Gating strategy for FACS ploidy sorting of an undifferentiated sarcoma for downstream RRBS profiling of ploidy sorted populations of cells. DAPI was measured using a 355 nm UV laser with a 450/50 bandpass filter. Ki-67 was measured using a 635 nm Red laser with a 670/30 bandpass filter. Forward scatter and side scatter were both measured from a 488nm blue laser on a linear scale. DAPI was also measured on a linear scale and was used to estimate DNA content per single cell. A control diploid cell line was used to establish accurate ploidy measurements prior to sorting. Forward vs. side scatter area was used to exclude debris, while the height vs area of the DAPI fluorescence was used to exclude doublets. FACS analysis revealed the presence of three major aberrant cell populations within our USARC, including a haploid population (1n), a nearly diploid population (2n, Ki-67 positive) and a WGD population (3n+).

## References

1 Sandve, G. K., Ferkingstad, E. & Nygard, S. Sequential Monte Carlo multiple testing. Bioinformatics 27, 3235-3241, doi:10.1093/bioinformatics/btr568 (2011).
