# Supplementary material for: Signatures of copy number alterations in human cancer
Source: Nature. 2022 Jun 15;606(7916):984–91. doi: 10.1038/s41586-022-04738-6 (PMC9242861; doi:10.1038/s41586-022-04738-6)
Supplement: Supplementary file 2 — Reporting Summary [file 41586_2022_4738_MOESM2_ESM.pdf]

nature research

Corresponding author(s): Nischalan Pillay and Ludmil Alexandrov

Last updated by author(s): 25/03/2022

Reporting Summary

Nature Research wishes to improve the reproducibility of the work that we publish. This form provides structure for consistency and transparency in reporting. For further information on Nature Research policies, see our Editorial Policies and the Editorial Policy Checklist.

Statistics

For all statistical analyses, confirm that the following items are present in the figure legend, table legend, main text, or Methods section.

n/a	Confirmed	
☐	☒	The exact sample size (*n*) for each experimental group/condition, given as a discrete number and unit of measurement
☐	☒	A statement on whether measurements were taken from distinct samples or whether the same sample was measured repeatedly
☐	☒	The statistical test(s) used AND whether they are one- or two-sided<br>*Only common tests should be described solely by name; describe more complex techniques in the Methods section.*
☐	☒	A description of all covariates tested
☐	☒	A description of any assumptions or corrections, such as tests of normality and adjustment for multiple comparisons
☐	☒	A full description of the statistical parameters including central tendency (e.g. means) or other basic estimates (e.g. regression coefficient) AND variation (e.g. standard deviation) or associated estimates of uncertainty (e.g. confidence intervals)
☐	☒	For null hypothesis testing, the test statistic (e.g. *F*, *t*, *r*) with confidence intervals, effect sizes, degrees of freedom and *P* value noted<br>*Give P values as exact values whenever suitable.*
☒	☐	For Bayesian analysis, information on the choice of priors and Markov chain Monte Carlo settings
☒	☐	For hierarchical and complex designs, identification of the appropriate level for tests and full reporting of outcomes
☐	☒	Estimates of effect sizes (e.g. Cohen's *d*, Pearson's *r*), indicating how they were calculated

Our web collection on statistics for biologists contains articles on many of the points above.

Software and code

Policy information about availability of computer code

Data collection	No software was used for data collection.
Data analysis	R v4.0.2<br>python v3.7.1<br>ASCAT v2.5.2 https://github.com/VanLoo-lab/ascat<br>ABSOLUTE v1.0.6<br>FACS DIVA v8.0.1<br>Beagle v5.1<br>alleleCounter v4.2.0<br>Python libraries:<br>SigProfilerExtractor v1.0.17 https://github.com/AlexandrovLab/SigProfilerExtractor<br>SigProfilerSingleSample v0.0.0.27 https://github.com/AlexandrovLab/SigProfilerSingleSample<br>SigProfilerMatrixGenerator v1.0 https://github.com/AlexandrovLab/SigProfilerMatrixGenerator<br>R libraries:<br>GenomicRanges v1.44.0<br>survival v3.2-11<br>survminer v0.4.6<br>qvalue v2.24.0<br>lsa v0.73.2<br>Rtsne v0.15<br>tidyr v1.1.3<br>ggplot2 v3.3.5<br>ggrepel v0.9.1

RColorBrewer v1.1-2
circlize v0.4.13
ComplexHeatmap v2.8.0
stringr v1.4.0
colorspace v2.0-2
seriation v1.3.0
dendextend v1.15.1
MASS v7.3-54
beanplot v1.2
corrplot v0.90
parallel v4.1.0
gtools v3.9.2
ABSOLUTE
ASCAT.sc v1.0
FACS DIVA v8.0.1
copynumber v1.26.0
Beagle v5.1
CAMDAC https://github.com/VanLoo-lab/CAMDAC
https://github.com/UCL-Research-Department-of-Pathology/panConusig
Other:
https://github.com/VanLoo-lab/ascat/tree/master/ReleasedData/TCGA_SNP6_hg19

For manuscripts utilizing custom algorithms or software that are central to the research but not yet described in published literature, software must be made available to editors and reviewers. We strongly encourage code deposition in a community repository (e.g. GitHub). See the Nature Research guidelines for submitting code & software for further information.

Data

Policy information about availability of data

All manuscripts must include a data availability statement. This statement should provide the following information, where applicable:

- Accession codes, unique identifiers, or web links for publicly available datasets
- A list of figures that have associated raw data
- A description of any restrictions on data availability

1. The TCGA ASCAT copy number profiles analysed here can be found at:
https://github.com/VanLoo-lab/ascat/tree/master/ReleasedData/TCGA_SNP6_hg19.
2. Exome sequencing data was accessed through dbGAP (phs000178.v11.p8) - https://dbgap.ncbi.nlm.nih.gov/
3. RRBS data for sorted ploidy populations can be accessed from the European Genome-Phenome Archive. Accession ID:EGAS00001006143
4. Single cell WGS data for sorted ploidy can be accessed from the European Genome-Phenome Archive. Accession ID:EGAS00001006144
5. Single cell models of chromothripsis WGS data were obtained from the European Nucleotide Archive Accession ID: PRJEB8037
6. TCGA clinical data was obtained from Integrated TCGA Pan-Cancer Clinical Data Resource - https://doi.org/10.1016/j.cell.2018.02.052
7. High confidence chromothripsis datasets were obtained from https://doi.org/10.1038/s41588-019-0576-7
8. Germline BRCA1/2 mutation data for TCGA samples were obtained from doi: 10.1093/jncics/pkz028
9. Li-Fraumeni data is deposited in the European Genome-Phenome Archive: Accession ID: EGAS00001005982
10. GRCh38 reference genome https://gatk.broadinstitute.org/hc/en-us/articles/360035890811-Resource-bundle
11. PCAWG chromothripsis calls https://www.nature.com/articles/s41586-020-1969-6
12. ECDNA calls across TCGA https://www.nature.com/articles/s41467-018-08200-y
13. SBS, DBS and ID signature exposures across TCGA https://www.nature.com/articles/s41586-020-1943-3
14. Smoking status of TCGA patients https://www.science.org/doi/10.1126/science.aag0299
15. Alcohol drinking status of TCGA patients https://www.nejm.org/doi/full/10.1056/nejmp1607591
16. Tandem duplicator phenotype evaluation across TCGA tumours https://www.sciencedirect.com/science/article/pii/S1535610818302654
17. COSMIC cancer gene census genes https://cancer.sanger.ac.uk/cosmic
18. Driver SNV and indel mutation calls https://www.sciencedirect.com/science/article/pii/S0092867417311364?via%3Dihub
19. Leucocyte counts were obtained from TCGA https://www.sciencedirect.com/science/article/pii/S1074761318301213?via%3Dihub
20. TCGA methylation data https://portal.gdc.cancer.gov/
21. TCGA gene expression data https://gdac.broadinstitute.org/
22. Gene expression derived hypoxia scores across TCGA https://www.nature.com/articles/s41588-018-0318-2
23. PCAWG rearrangement classes https://www.sciencedirect.com/science/article/pii/S0092867420309971
24. HPV testing status from TCGA head and neck cancers https://genomemedicine.biomedcentral.com/articles/10.1186/gm453

Field-specific reporting

Please select the one below that is the best fit for your research. If you are not sure, read the appropriate sections before making your selection.

☒ Life sciences ☐ Behavioural & social sciences ☐ Ecological, evolutionary & environmental sciences

For a reference copy of the document with all sections, see nature.com/documents/nr-reporting-summary-flat.pdf

Life sciences study design

All studies must disclose on these points even when the disclosure is negative.

Sample size	12,241 TCGA samples were analysed from which copy number profiles were generated for 9,873 cancers and matching germline DNA of 33 different cancer types. Additionally, a set of whole-genome sequences from 512 cancers of the International Cancer Genome Consortium (ICGC) that overlapped with tumour profiles in TCGA were analysed to generate WGS-derived copy number profiles. Whole-exome sequences from 282 cancers from TCGA was analysed to generate exome-derived copy number profiles.<br>RRBS sample - 5 ploidy cell fractions from one patient tumour sample was used.<br>Single cell DNA sequencing - 502 single cells from one patient tumour sample.
Data exclusions	Samples with poor ploidy/purity fits, mismatches to germline data and over-segmentation through the copy number profiling were excluded.
Replication	To evaluate generalizability across platforms. A set of samples from TCGA with both SNP-array and exome sequencing data were selected (n=282). For whole-genome sequencing data, we examined 512 whole-genome sequenced samples from the PCAWG project overlapping with TCGA samples with microarray data. We also systematically examined copy number signatures derived from WGS, WES and SNP6 profiles of the same samples which demonstrated a strong concordance between signatures identified through different platforms (median cosine similarity>0.8).
Randomization	No randomisation of samples was performed. For signatures, simulations of copy number profiles incorporating processes of chromothripsis, whole-genome doubling, and chromosomal duplication were performed.The outline of various bootstrapping methods and simulations are provided in detail in the Methods section.
Blinding	No blinding was performed as there were no relevant treatment arms.

Reporting for specific materials, systems and methods

We require information from authors about some types of materials, experimental systems and methods used in many studies. Here, indicate whether each material, system or method listed is relevant to your study. If you are not sure if a list item applies to your research, read the appropriate section before selecting a response.

Materials & experimental systems

n/a	Involved in the study	
☒	☐	Antibodies
☒	☐	Eukaryotic cell lines
☒	☐	Palaeontology and archaeology
☒	☐	Animals and other organisms
☐	☒	Human research participants
☒	☐	Clinical data
☒	☐	Dual use research of concern

Methods

n/a	Involved in the study	
☒	☐	ChIP-seq
☐	☒	Flow cytometry
☒	☐	MRI-based neuroimaging

Human research participants

Policy information about studies involving human research participants

Population characteristics	All anonymised patient data used in this study has been previously published by the TCGA and is available through publicly accessible repositories. Samples used for RRBS sequencing and single cell DNA sequencing were obtained from patients with informed consent.
Recruitment	Not applicable.
Ethics oversight	Informed consent from patients and ethical approval for tissue biobanking was obtained through the UCL/UCLH Biobank for Studying Health and Disease (REC reference: 20/YH/0088 - NHS Health Research Authority). Approval for the study and ethics oversight was granted by NHS Health Research Authority (REC reference: 16/NW/0769).

Note that full information on the approval of the study protocol must also be provided in the manuscript.

Flow Cytometry

Plots

Confirm that:

☒ The axis labels state the marker and fluorochrome used (e.g. CD4-FITC).

☒ The axis scales are clearly visible. Include numbers along axes only for bottom left plot of group (a 'group' is an analysis of identical markers).

☒ All plots are contour plots with outliers or pseudocolor plots.

☒ A numerical value for number of cells or percentage (with statistics) is provided.

Methodology

Sample preparation	Fresh frozen tumour tissue was thawed on ice, dissected, and homogenized with 500 µl of lysis buffer (NUC201-1KT, Sigma). Following the release of single nuclei, samples were centrifuged, and the resulting precipitate removed. A 10 µl sample was taken to count and evaluate the extracted nuclei. The lysate was cleaned using a sucrose gradient following the manufacturer's instructions (NUC201-1KT, Sigma). After cleaning, the nuclei were centrifuged at 800g for 5-10 min at 4C and resuspended in PBS, supplemented with 140µg/ml RNase (19101 Qiagen) and stained with 1µg/ml DAPI (Sigma-Aldrich), as well as 2.5µg/ml Ki-67 Antibody (Biolegend UK LTD) per 1 million cells in 100 µl.
Instrument	Aria Fusion cell sorter (BD bioscience, San Jose, CA, USA)
Software	BD FACSDiva 8.0.1 and flowCore version 2.6.0 (Bioconductor package)
Cell population abundance	Single cell sorting: Total population=171,664, Gate1 (FSC,SSC-124,723 events), Gate 2 (DAPI - 89,954 events), Gate 3 (DAPI vs Ki67: haploid-15,522, diploid/Ki67 high=34,741 events, diploid/Ki67 low=4,236 events,tetraploid=19,934 events).<br>Ploidy sorting for RRBS: Total population=455,072, Gate1 (FSC,SSC-244,186 events), Gate 2 (DAPI - 101,578 events), Gate 3 (DAPI vs Ki67: haploid-24,035 events , diploid-30,974 events ,tetraploid=10,991 events).
Gating strategy	Stained nuclei were analysed using a FACS Aria Fusion cell sorter (BD bioscience, San Jose, CA, USA) on FACS DIVA software v8.0.1. Cells were sorted using a 130 micron nozzle with 12psi set for sheath pressure. Each gated population of interest was collected into a separate 1.5ml tube and a custom sort precision of 0-16-0 (Yield-Purity-Phase) was used. For cells collected into plates, the sort precision used was Purity, defined as 32-32-0 (Yield-Purity-Phase). DAPI was measured using a 355 nm UV laser with a 450/50 bandpass filter. Ki-67 was measured using a 635 nm Red laser with a 670/30 bandpass filter. Forward scatter and side scatter were both measured from a 488nm blue laser on a linear scale. DAPI was also measured on a linear scale and was used to estimate DNA content per single cell. A control diploid cell line was used to establish accurate ploidy measurements prior to sorting. Forward vs. side scatter area was used to exclude debris, while the height vs area of the DAPI fluorescence was used to exclude doublets. FACS analysis revealed the presence of three major aberrant cell populations within our USARC, including a haploid population (1n), a nearly diploid population (2n, Ki-67 positive) and a WGD population (3n+). A non-proliferating, non-aberrant, normal cell population was also identified (2n, Ki-67 negative).

☒ Tick this box to confirm that a figure exemplifying the gating strategy is provided in the Supplementary Information.
